# MFSD4A inhibits the malignant progression of nasopharyngeal carcinoma by targeting EPHA2

**DOI:** 10.1038/s41419-022-04793-x

**Published:** 2022-04-11

**Authors:** Huiyun Yang, Guanjie Qin, Zan Luo, Xiangyun Kong, Chunqiao Gan, Ruyun Zhang, Wei Jiang

**Affiliations:** 1 Department of Oncology, Xiangya Hospital, Central South University, Changsha, 410008 China; 2 Department of Radiation Oncology, Guilin Medical University Affiliated Hospital, Guilin, 541001 China

**Keywords:** Tumour-suppressor proteins, Head and neck cancer, Prognostic markers

## Abstract

DNA Methylation can lead to abnormal gene expression. In the present study, we investigated whether the expression of methylated *MFSD4A* (major facilitator superfamily domain containing 4 A) was downregulated in nasopharyngeal carcinoma (NPC) and whether it is associated with malignant progression and poor prognosis of NPC. Bioinformatic analysis, bisulfite pyrosequencing, quantitative real-time reverse transcription PCR, and western blotting assays were performed to explore the relationship between hypermethylation of *MFSD4A* and its expression in NPC. The role of MFSD4A in NPC was verified by Cell Cycle Kit 8, transwell assays and flow cytometry in vitro and by animal experiments in vivo. Mass spectrometry, co-immunoprecipitation, and immunofluorescence assays were applied to explore the mechanism by which MFSD4A inhibits NPC. The prognostic significance of MFSD4A or EPHA2 was investigated by immunohistochemical analysis of clinical specimens. Hypermethylation of the promoter region of *MFSD4A* led to decreased expression of *MFSD4A*. When *MFSD4A* expression was upregulated or downregulated, the proliferation, apoptosis, migration, and invasion abilities of NPC cells were altered accordingly. Mechanistically, MFSD4A could specifically bind to and degrade EPH receptor A2 (EPHA2) by recruiting ring finger protein 149 (RNF149), which led to alterations in the EPHA2-mediated PI3K-AKT-ERK1/2 pathway and epithelial-mesenchymal transition (EMT), thereby affecting NPC progression. Clinically, high MFSD4A expression or low-EPHA2 expression was associated with better prognosis for patients with NPC. In all, reduced *MFSD4A* expression in NPC is caused by promoter hypermethylation. MFSD4A or EPHA2 expression is associated with the malignant biological behavior and prognosis of NPC. MFSD4A is a promising potential therapeutic target for NPC.

## Introduction

Nasopharyngeal carcinoma (NPC) is one of the most common malignancies and has a high incidence in Southeast Asia and North Africa (especially in Southern China) [1, 2]. However, 20%–30% of patients develop residual recurrence of local tumor lesions or distant metastases after standard treatment, representing the main reason for treatment failure of NPC [3]. Therefore, in addition to preventing the malignant progression of NPC, exploring the molecular mechanisms of recurrence and metastasis, and finding new therapeutic targets and relevant prognostic molecular indicators has become a challenge and an opportunity to improve the survival rate of NPC.

DNA methylation is an epigenetic phenomenon allowing the regulation of gene transcription and expression, which is closely related to the occurrence and development of tumors [4–6]. In our previous study, high-throughput sequencing showed that methylation occurs in many genes in NPC tissues compared with that in normal nasopharyngeal tissues, and the methylation levels showed prognostic significance [7]. Our further studies clarified the role of some aberrantly methylated genes in tumor progression, such as *ZNF154* (encoding zinc finger protein 154) and *SFRP1* (encoding secreted frizzled related protein 1), which act as antioncogenes to inhibit NPC metastasis [8, 9]. Other researchers discovered that methylation-modified *PCDH10* (encoding protocadherin 10) and *SOX11* (encoding SRY-box transcription factor 11) can inhibit the proliferation and invasive ability of NPC cells [10, 11]. Thus, determining the methylation status of genes is very important to further understand the mechanism of tumor development.

Major facilitator superfamily domain containing 4 A (MFSD4A) is a member of the major facilitator superfamily (MFS) [12], and is responsible for the transportation of substances such as monosaccharides, polysaccharides, amino acids, and peptides [13, 14]. However, few studies have investigated the function of MFSD4A, let alone in cancer. Kanda et al. first reported the methylation of *MFSD4A* and showed that MFSD4A could inhibit the malignant phenotype of gastric cancer cells [15]. Regrettably, in addition to a lack of in vivo assays, that study also failed to clarify the mechanism of action of MFSD4A. Therefore, the present study aimed to address the gap in the field regarding the anti-cancer effect of MFSD4A and the mechanisms involved. In our study, we found that *MFSD4A* expression was regulated by methylation of its promoter region in NPC and that MFSD4A could degrade EPH receptor A2 (EPHA2) by recruiting ring finger protein 149 (RNF149), leading to the suppression of the phosphatidylinositol-4,5-Bisphosphate 3-Kinase (PI3K)/protein kinase B (AKT)/extracellular regulated kinase 1/2 (ERK1/2) pathway and epithelial-mesenchyme transition (EMT), thereby inhibiting the proliferation, invasion, and migration of NPC cells. In addition, we found that low expression of MFSD4A was related to poor prognosis for patients with NPC via immunohistochemical analysis. Thus, exploration of the role of MFSD4A in NPC might provide new insights into tumor targeting therapy.

## Results

### Hypermethylation of the promoter region of *MFSD4A* in NPC

**Fig. 1:**
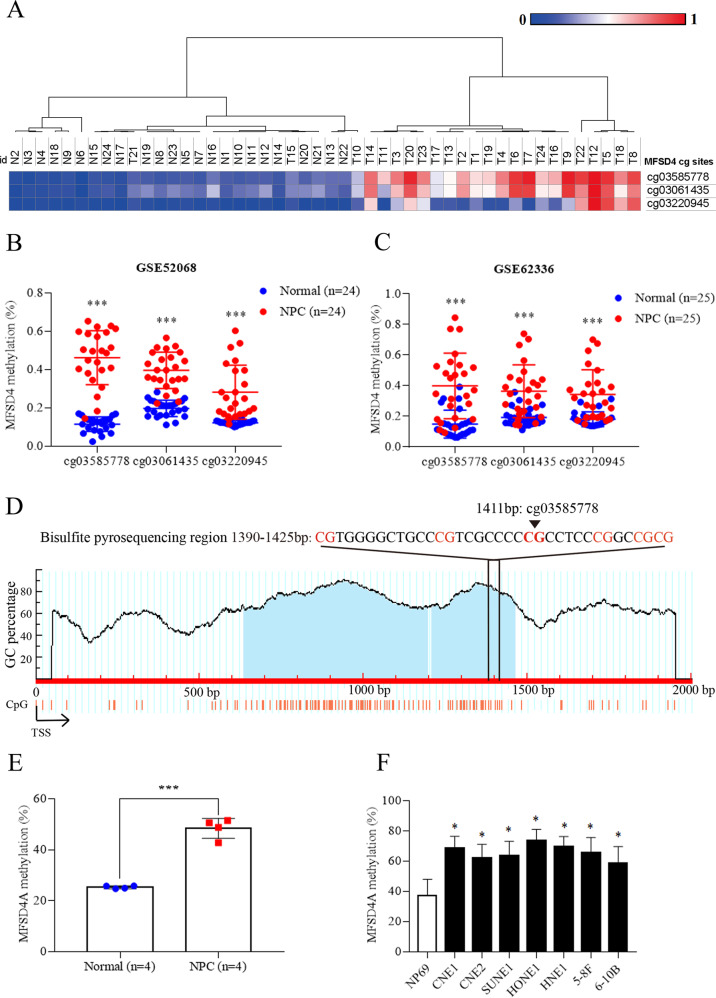
*MFSD4A* is hypermethylated in nasopharyngeal carcinoma. **A** Heatmap cluster of *MFSD4A* CG sites between NPC (*n* = 24) and normal nasopharyngeal tissue samples (*n* = 24). The methylation level of *MFSD4A* CG sites in the GSE52068 (**B**) and GSE62336 (**C**) microarray data between NPC and normal nasopharyngeal tissue samples. **D** Schematic of CpG islands and bisulfite pyrosequencing regions in the *MFSD4A* promoter. Red region, input sequence; Blue region, CpG islands; TSS, transcription start site; cg03585778: a CG site of *MFSD4A* identified in GSE52068; red text: CG sites for bisulfite pyrosequencing; bold red text, the most methylated CG sites in *MFSD4A*. (**E**, **F**) The methylation levels of the *MFSD4A* promoter region defined by bisulfite pyrosequencing analysis in normal (*n* = 4) and NPC (*n* = 4) tissues (**E**), and in NP69 and NPC cell lines (CNE1, CNE2, SUNE1, HONE1, HNE1, 5-8 F, and 6-10B) (**F**). *P < 0.05, ***P < 0.001.

In the dataset (GSE52068), the methylation frequency of *MFSD4A* in 24 pairs of NPC and normal nasopharyngeal tissues are shown in Fig. 1A. Three CpG sites in *MFSD4A* (cg03585778, cg03061435, and cg03220945) were significantly hypermethylated in NPC but not in normal nasopharyngeal tissues (Fig. 1B), which was verified in another The Cancer Genome Atlas (TCGA) dataset (GSE62336) (Fig. 1C). In addition, bisulfite pyrosequencing analysis was performed for cg03585778, which was the most significantly hypermethylated CpG site (Fig. 1D). The results proved that, whether in NPC tissues (Fig. 1E) or NPC cell lines (Fig. 1F), a high methylation level at this region was observed in *MFSD4A*. Based on the above findings, we concluded that the promoter region of *MFSD4A* was hypermethylated in NPC, compared with that in normal tissues.

### Hypermethylation leads to the decreased expression of *MFSD4A*

**Fig. 2:**
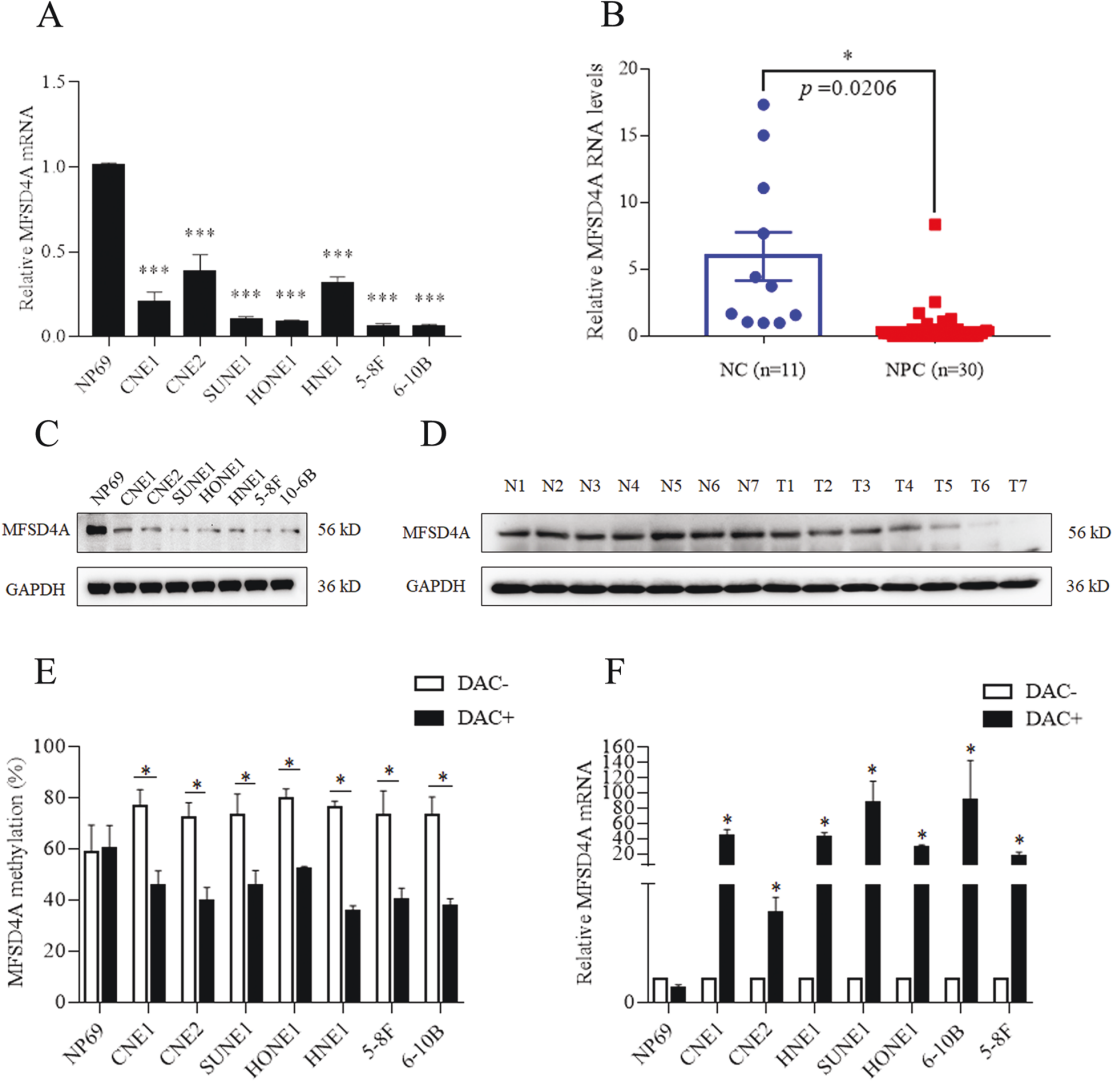
Promoter hypermethylation could downregulate MFSD4A expression in nasopharyngeal carcinoma. qRT-PCR analysis of *MFSD4A* mRNA expression in normal nasopharyngeal epithelial cell lines (NP69) and NPC cell lines (CNE1, CNE2, SUNE1, HONE1, HNE1, 5-8 F, and 6-10B) (**A**), in normal (*n* = 11) and NPC (*n* = 30) tissues (**B**). MFSD4A and GAPDH protein levels in NP69 and NPC cell lines (**C**), in normal (N, *n* = 7) and NPC (T, *n* = 7) tissues (**D**). **E** *MFSD4A* promoter methylation levels determined by bisulfite pyrosequencing analysis in NP69 and NPC cell lines treated with or without DAC. **F** qRT-PCR analysis of MFSD4A mRNA expression in NP69 and NPC cell lines treated with or without DAC. *P < 0.05, ***P < 0.001.

The expression of MFSD4A was explored. The mRNA levels of *MFSD4A* in NPC cell lines (Fig. 2A) and NPC tissues (Fig. 2B) were lower than those in NP69 cells and normal nasopharyngeal epithelial tissues, respectively. The protein levels of MFSD4A were also higher in NP69 cells (Fig. 2C) and normal tissues (Fig. 2D). When 5-aza-2ʹ-deoxycytidine (DAC) (an inhibitor of methyltransferase) was added, not only was the methylation of *MFSD4A* reduced (Fig. 2E), but also the expression of *MFSD4A* was increased (Fig. 2F) in NPC cell lines, which indicated that methylation of *MFSD4A* could cause a decrease in the expression of *MFSD4A*.

### MFSD4A affects the phenotype and signaling pathway of NPC cells

**Fig. 3:**
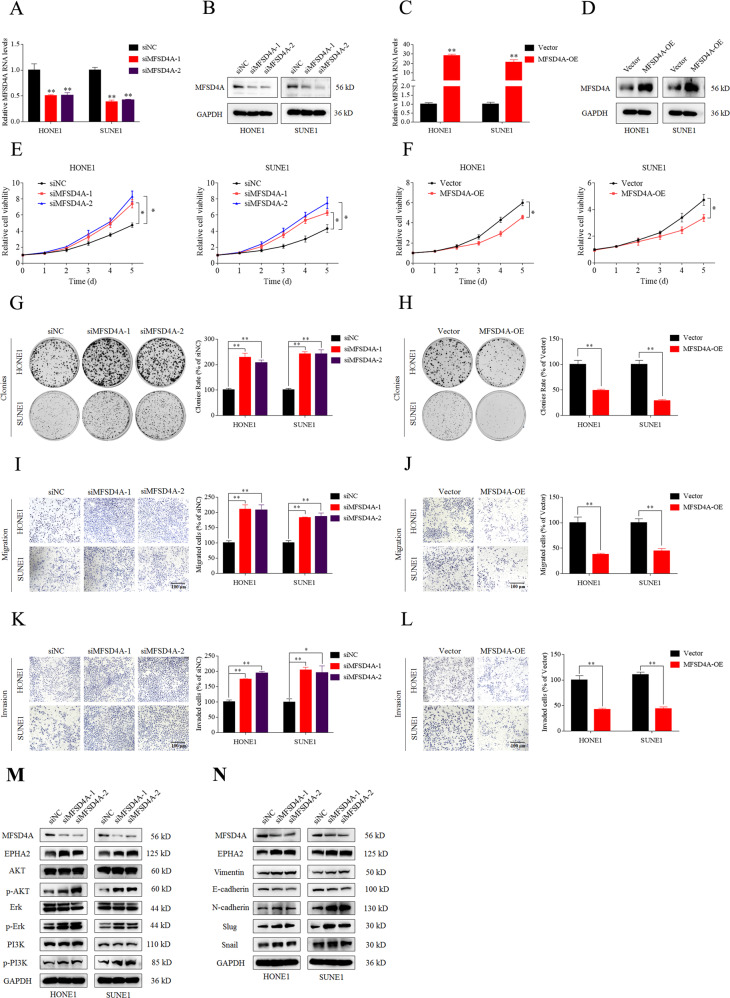
MFSD4A inhibits the proliferation, migration, and invasion of NPC cells in vitro. **A** qRT-PCR analysis of *MFSD4A* mRNA in SUNE1 and HONE1 cells transfected with vector or siMFSD4A. **B** Immunoblot analysis of MFSD4A and GAPDH in SUNE1 and HONE1 cells transfected with empty vector or siMFSD4A. **C** qRT-PCR analysis of *MFSD4A* mRNA in SUNE1 and HONE1 cells transfected with vector or the MFSD4A overexpression plasmid. **D** Immunoblot analysis of MFSD4A and GAPDH in SUNE1 and HONE1 cells transfected with vector or the MFSD4A overexpression plasmid. Downregulation of *MFSD4A* by siMFSD4A promoted cell proliferation (**E**) while upregulation of *MFSD4A* inhibited cell proliferation (**F**) in HONE1 and SUNE1 cells, as assessed using CCK-8 assays. Downregulation of *MFSD4A* by siMFSD4A promoted cell proliferation (**G**) while upregulation of *MFSD4A* inhibited cell proliferation (**H**) in HONE1 and SUNE1 cells, as assessed usng colony formation assays (left); the colonies were counted and compared using a *t*-test (right). Downregulation of *MFSD4A* by siMFSD4A promoted cell migration (**I**), while upregulation of *MFSD4A* inhibited cell migration (**J**) in HONE1 and SUNE1 cells, as assessed using Transwell assays without Matrigel (left); the cells were counted and compared using a *t*-test (right). Downregulation of *MFSD4A* by siMFSD4A promoted invasion (**K**), while upregulation of *MFSD4A* inhibited cell invasion (**L**) in HONE1 and SUNE1 cells, as assessed using Transwell assays with Matrigel (left); the cells were counted and compared using a *t*-test (right). **M** Downregulation of *MFSD4A* by siMFSD4A increased EPHA2 protein levels and promoted the PI3K-AKT-ERK1/2 pathway. **N** Downregulation of *MFSD4A* by siMFSD4A increased EPHA2 expression and promote EMT. *P < 0.05, **P < 0.01.

The role of MFSD4A in NPC has not been reported. Our study confirmed the anticancer effect of MFSD4A in NPC. The siRNAs targeting *MFSD4A* (siF-1 and siF-2) and siRNA-vector (siNC) were transfected transiently to silence *MFSD4A* (Fig. 3A, B) while *MFSD4A* overexpression plasmids (MFSD4A-OE) were used to overexpress MFSD4A mRNA and protein in HONE1 and SUNE1 cells (Fig. 3C, D). CCK-8 assays suggested that NPC cells with downregulated or upregulated MFSD4A expression displayed increased (Fig. 3E, *P* < 0.05) or decreased proliferation (Fig. 3F, *P* < 0.05), respectively. Similar conclusion was drawn from plate clone formation assays. When the expression of *MFSD4A* was downregulated or upregulated, the number of clones increased (Fig. 3G) or decreased (Fig. 3H) accordingly. Transwell assays were also performed. NPC cells acquired stronger or weaker migration and invasion abilities when *MFSD4A* expression was downregulated (Fig. 3I, K) or upregulated (Fig. 3J, L), respectively. Downregulation of *MFSD4A* by siMFSD4A increased EPHA2 protein levels, activated the PI3K-AKT-ERK1/2 pathway, as evidenced by increased levels of phosphorylated members of the pathway (Fig. 3M), and promoted EMT, as evidenced by decreased levels of epithelial proteins, and increased levels of mesenchymal proteins (Fig. 3N). In addition, the overexpression of MFSD4A also promoted apoptosis in NPC cells (Fig. S1).

### MFSD4A binds to EPHA2 and RNF149

**Fig. 4:**
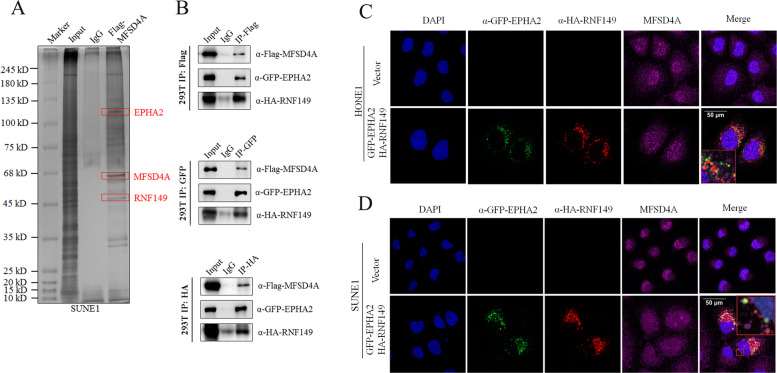
MFSD4A interacts with EPHA2 and RNF149. **A** Immunoprecipitation (with anti-Flag antibodies or IgG) and SDS PAGE analysis of SUNE1 cells stably transfected with FLAG-MFSD4A. **B** Immunoprecipitation (with anti-Flag antibodies or IgG) and immunoblot analysis (with anti-GFP, anti-FLAG, and anti-HA antibodies) of 293 T cells transfected with plasmids encoding FLAG-MFSD4A, GFP-EPHA2, and HA-RNA149 (upper). Immunoprecipitation (with anti-GFP antibodies or IgG) and immunoblot analysis (with anti-GFP, anti-FLAG, and anti-HA antibodies) of 293 T cells transfected with plasmids encoding FLAG-MFSD4A, GFP-EPHA2, and HA-RNA149 (middle). Immunoprecipitation (with anti-HA antibodies or IgG) and immunoblot analysis (with anti-GFP, anti-FLAG, and anti-HA antibodies) of 293 T cells transfected with plasmids encoding FLAG-MFSD4A, GFP-EPHA2, and HA-RNA149 (lower). (C-D) MFSD4A could bind to GFP-EPHA2 and HA-RNF149 simultaneously in HONE1 (**C**) and SUNE1 (**D**) cells transfected with plasmids encoding GFP-EPHA2 and HA-RNF149, as assessed using immunofluorescence assays.

Co-IP and mass spectrometry analysis were applied to explore the molecular mechanism of cancer inhibition by MFSD4A. Co-IP and mass spectrometry analysis showed that MFSD4A bound to EPHA2 and RNF149 (Fig. 4A, Supplementary Fig. 2). To further confirm this observation, we conducted co-IP assays in 293 T cells. Flag-tagged MFSD4A pulled down GFP-tagged EPHA2 and HA-tagged RNF149 (Fig. 4B: upper), GFP-tagged EPHA2 pulled down Flag-tagged MFSD4A and HA-RNF149 (Fig. 4B: middle), and HA-tagged RNF149 pulled down Flag-tagged MFSD4A and GFP-tagged EPHA2 (Fig. 4B: lower). Immunofluorescence assays also confirmed the mutual binding (colocalization) among MFSD4A, EPHA2, and RNF149 in HONE1 (Fig. 4C) and SUNE1 cells (Fig. 4D). According to the immunofluorescence results, MFSD4A was distributed in the nucleus and cytoplasm, and EPH2A and RNF149 were mainly distributed in the cytoplasm. MFSD4A could bind to EPHA2 and RNF149 mainly in the cytoplasm.

### MFSD4A promotes ubiquitination and degradation of EPHA2 via RNF149

**Fig. 5:**
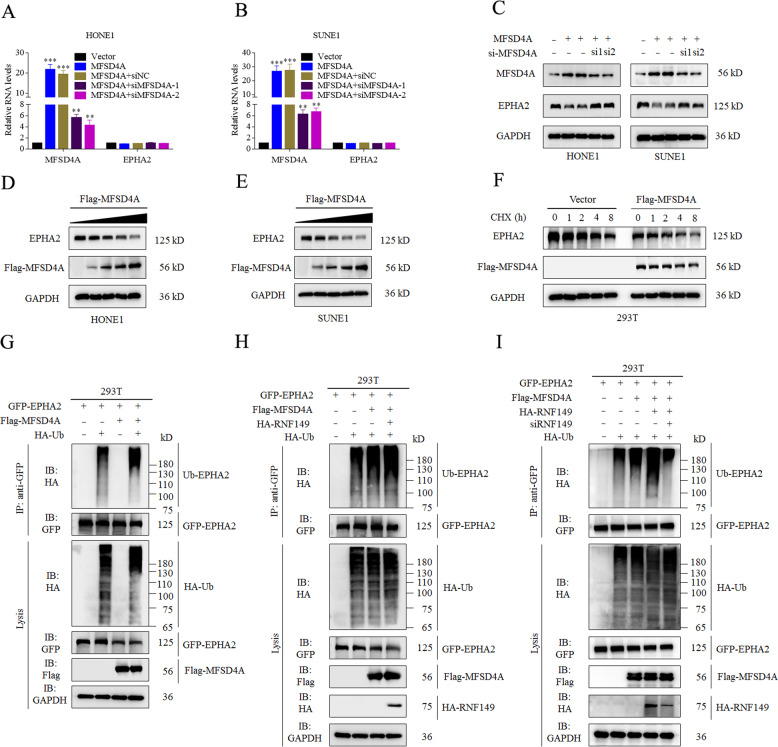
MFSD4A promotes ubiquitination and degradation of EPHA2 via RNF149. Altered expression of MFSD4A did not affect the mRNA levels of *EPHA2* in HONE1 (**A**) and SUNE1 (**B**) cells. **C** Overexpression or inhibition of MFSD4A was followed by a decrease or increase in EPHA2 protein levels. Immunoblot analysis (with anti-Flag, EPHA2, and GAPDH antibodies) in HONE1 (**D**) and SUNE1 (**E**) cells transfected with various doses of Flag-MFSD4A (0, 0.25, 0.5, 1, and 2 μg). **F** Immunoblot analysis of Flag-MFSD4A, EPHA2, and GAPDH in 293 T cells transfected with the empty vector, or Flag-MFSD4A for 24 h, followed by treatment with CHX for 0–8 h. **G** Immunoprecipitation (with anti-GFP antibodies) and immunoblot analysis (with anti-GFP, anti-HA, anti-Flag, or anti-GAPDH antibodies) of 293 T cells transfected with plasmids encoding FLAG-MFSD4A, HA-Ubiquitin, GFP-EPHA2, and empty vector for 24 h. **H** Immunoprecipitation (with anti-GFP antibodies) and immunoblot analysis (with anti-GFP, anti-HA, anti-Flag, or anti-GAPDH antibodies) of 293 T cells transfected with plasmids encoding FLAG-MFSD4A, HA-Ubiquitin, HA-RNF149, GFP-EPHA2, and empty vector for 24 h. **I** Immunoprecipitation (with anti-GFP antibodies) and immunoblot analysis (with anti-GFP, anti-HA, anti-Flag, or anti-GAPDH antibodies) of 293 T cells transfected with plasmids encoding FLAG-MFSD4A, HA-Ubiquitin, HA-RNF149, siRNF149, GFP-EPHA2, and empty vector for 24 h.**P < 0.01, ***P < 0.01.

When MFSD4A was up or downregulated, the *EPHA2* mRNA level remained unchanged in HONE1 (Fig. 5A) and SUNE1 cells (Fig. 5B); however, the EPHA2 protein level decreased or increased, respectively (Fig. 5C). Moreover, when we transfected different doses of MFSD4A plasmids (0, 0.25, 0.5, 1, and 2 μg) (Fig. 5D and E) into NPC cells to change the expression of *MFSD4A*, the protein levels of EPHA2 also varied in HONE1 (Fig. 5D) and SUNE1 cells (Fig. 5E). Overexpression of *MFSD4A* increased the degradation of EPHA2 in the presence of cycloheximide (CHX, used to block *de novo* protein synthesis) in 293 T cells (Fig. 5F). How does MFSD4A degrade EPHA2? Through a literature review, we found that EPHA2 could be degraded by ubiquitinating enzymes [16]; however, MFSD4A is not a ubiquitinating enzyme and thus might not degrade EPHA2 directly. As reported above (Fig. 4B), MFSD4A and EPHA2 also bind RNF149, which is a ubiquitin ligase. Does MFSD4A mediate the degradation of EPAH2 via RNF149? RNF149, as an E3 ligase, is involved in protein ubiquitination [17], which causes proteasome-mediated degradation of substrate proteins [18, 19]. To clarify the relationship between RNF149 and EPHA2 ubiquitination, we conducted co-IP assays and found that overexpression of *MFSD4A* could promote the ubiquitination of EPHA2 (Ub-EPHA2) in 293 T cells (Fig. 5G), and when MFSD4A and RNF149 in 293 T cells were elevated at the same time, ubiquitination of EPHA2 was more pronounced (Fig. 5H). When RNF149 was silenced, the ubiquitination of EPHA2 was also diminished (Fig. 5I). Therefore, we hypothesized that overexpression of *MFSD4A* promoted the ubiquitination of EPHA2 by recruiting RNF149, leading to degradation of EPAH2.

### MFSD4A inhibits tumor progression via EPHA2

**Fig. 6:**
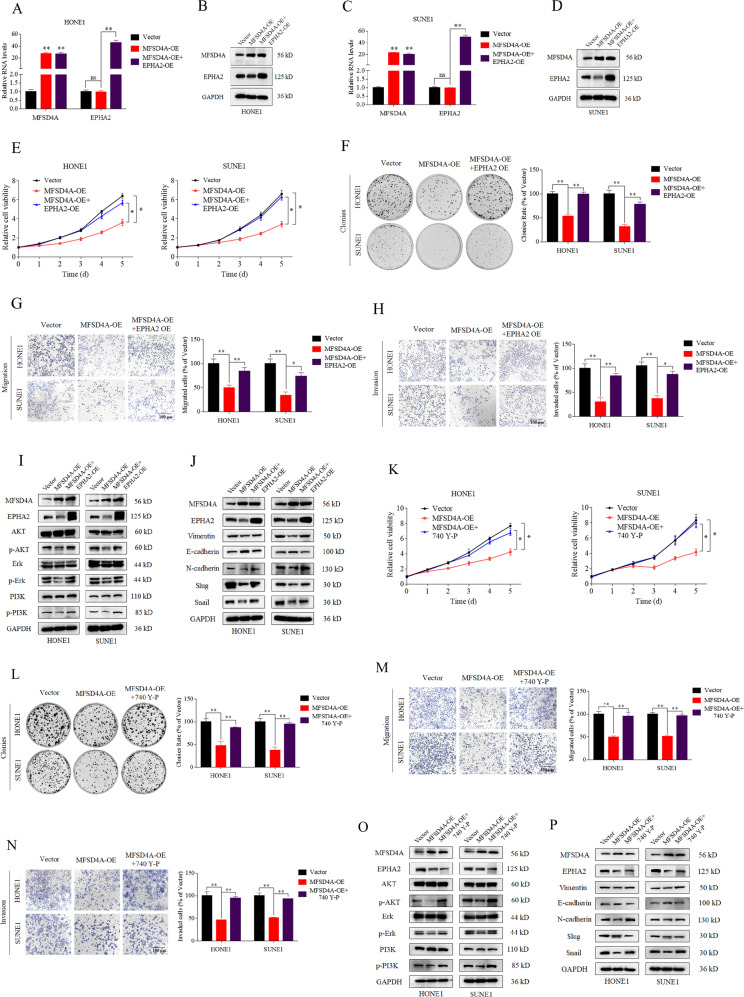
EPHA2 reversed the proliferation, migration, invasion, PI3K-AKT-ERK1/2 pathway activation, and EMT induced by upregulated MFSD4A. EPHA2 was upregulated by transient transfection in HONE1 cells overexpressing MFSD4A, which was detected using qRT-PCR (**A**) and western blotting (**B**). EPHA2 was upregulated by transient transfection in SUNE1 cells overexpressing MFSD4A, which was detected using qRT-PCR (**C**) and western blotting (**D**). **E** EPHA2 reversed the proliferation induced by upregulated MFSD4A, as demonstrated by CCK-8 assays in HONE1 and SUNE1 cells. **F** EPHA2 reversed the proliferation induced by upregulated MFSD4A, as demonstrated by colony formation assays in HONE1 and SUNE1 cells. EPHA2 reversed the migration (**G**) and invasion (**H**) induced by upregulated MFSD4A, as validated by Transwell assays in HONE1 and SUNE1 cells. Cells in the Transwell assays were calculated and compared, and the data are presented using histograms (**G**, **H**, right). EPHA2 reversed PI3K-AKT-ERK1/2 pathway activation (**I**) and EMT (**J**) as demonstrated using immunoblot analysis. *P < 0.05, **P < 0.01. (K)740Y-P (PI3K activator) reversed the proliferation induced by upregulated MFSD4A, as demonstrated by CCK-8 assays in HONE1 and SUNE1 cells. **L** 740Y-P (PI3K activator) reversed the proliferation induced by upregulated MFSD4A, as demonstrated by colony formation assays in HONE1 and SUNE1 cells. (M-N) 740Y-P (PI3K activator) reversed the migration (**M**) and invasion (**N**) induced by upregulated MFSD4A, as validated by Transwell assays in HONE1 and SUNE1 cells. Cells in the Transwell assays were calculated and compared, and the data are presented using histograms (**M**, **N**, right). 740Y-P (PI3K activator) reversed PI3K-AKT-ERK1/2 pathway activation (**O**) and EMT (**P**) as demonstrated using immunoblot analysis.

Whether MFSD4A inhibits the malignant progression of NPC via EPHA2 required further demonstration. On the background of MFSD4A overexpression, we increased *EPHA2* expression by transfection of EPHA2 plasmids into HONE1 (Fig. 6A, B) and SUNE1 cells (Fig. 6C, D). CCK-8 (Fig. 6E) and colony formation assays (Fig. 6F) indicated that the proliferation of NPC cells was reactivated and the migration and invasion abilities of NPC cells inhibited by MFSD4A were rescued by *EPHA2* overexpression, according to Transwell assays (Fig. 6G: migration; Fig. 6H: invasion). In addition, overexpression of EPHA2 activated the PI3K-AKT-ERK1/2 pathway (Fig. 6I), EMT (Fig. 6J) and inhibited apoptosis (Fig. S1). These results verified that EPHA2 reversed the inhibition of tumor progression, signaling pathways, EMT and promotion of apoptosis induced by *MFSD4A* overexpression. Further, the PI3K activator (740 Y-P) also activated the proliferation (Fig. 6K, L), migration (Fig. 6M), invasion (Fig. 6N), PI3K-AKT-ERK1/2 pathway (Fig. 6O), and EMT (Fig. 6P) inhibited by the overexpression of MFSD4A.

### MFSD4A suppresses the malignant progression of NPC in vivo

**Fig. 7:**
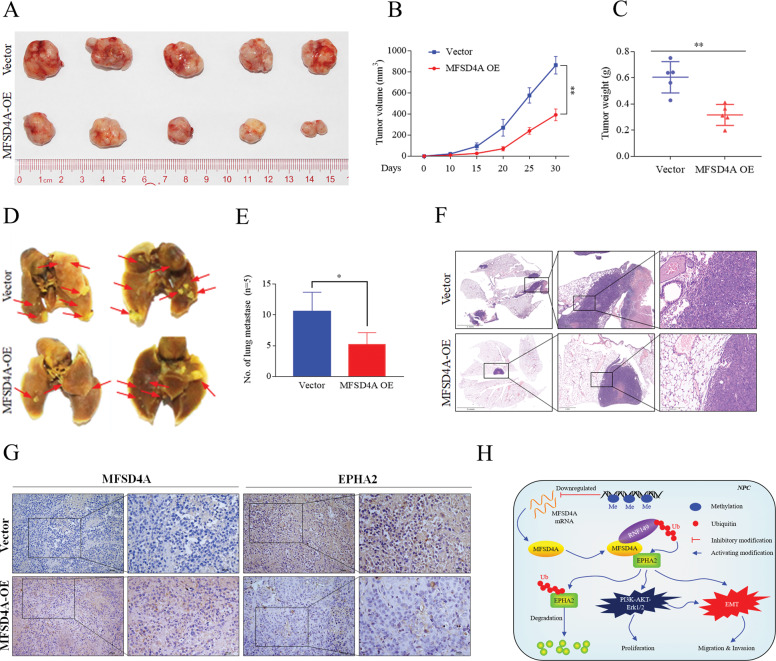
Animal experiments and a diagram of the model mechanism. **A**, **B** Tumor growth models. **A** The tumor volume of mice was reduced after overexpression of MFSD4A (**B**). **C** Tumor weight was reduced in mice after overexpression of MFSD4A. **D** Mouse lung metastasis models. The red arrows represented the metastatic areas. **E** The number of pulmonary metastases of mice was compared between mice overexpressing MFSD4A and the control group. **F** Hematoxylin-eosin staining assays demonstrating that overexpression of MFSD4A induced fewer lung metastases compared with those in the control group. **G** Immunohistochemistry assays showing the expression of MFSD4A (left) and EPHA2 (right) in mouse tissues overexpressing MFSD4A and the control group. **H** Schematic diagram of the mechanism of MFSD4A in NPC. *P < 0.05, **P < 0.01.

Tumor growth and metastasis models in mice were constructed to verify the anti-cancer effect of MFSD4A in vivo. NPC cells overexpressing *MFSD4A* were injected into mice. NPC cells overexpressing *MFSD4A* formed smaller (Fig. 7A, B) and lighter tumors (Fig. 7C), and caused fewer lung metastases (Fig. 7D–F) compared with mice bearing wild-type tumors. Immunohistochemical assays indicated that overexpression of *MFSD4A* (Fig. 7G, left) was accompanied by a decrease in EPHA2 levels (Fig. 7G, right). A map of the molecular mechanism is presented in Fig. 7H.

### Immunohistochemistry-based risk stratification

**Fig. 8:**
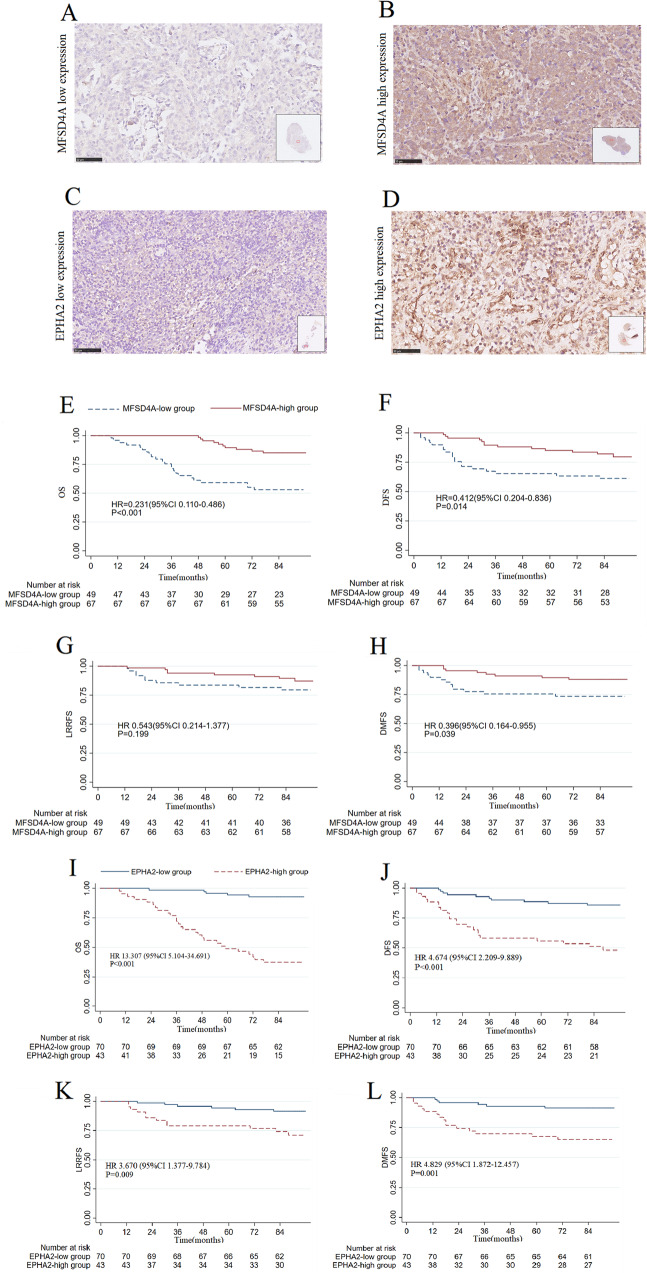
Immunohistochemistry-based risk stratification and prognostic analysis of MFSD4A or EPHA2. The immunohistochemical staining of a sample from a patient in the MFSD4A-low group (**A**) and from a patient in the MFSD4A-high group (**B**). The immunohistochemical staining of a sample from a patient in the EPHA2-low group (**C**) and from a patient in the EPHA2-high group (**D**). **E** Comparison of overall survival (OS) between the MFSD4A-high group and the MFSD4A-low group. **F** Comparison of disease-free survival (DFS) between the MFSD4A-high group and the MFSD4A-low group. **G** Comparison of local-regional relapse-free survival (LRRFS) between the MFSD4A-high group and the MFSD4A-low group. **H** Comparison of distant metastasis-free survival (DMFS) between the MFSD4A-high group and the MFSD4A-low group. **I** Comparison of overall survival (OS) between the EPHA2-high group and the EPHA2-low group. **J** Comparison of disease-free survival between the EPHA2-high group and the EPHA2-low group. **K** Comparison of local-regional relapse-free survival (LRRFS) between the EPHA2-high group and the EPHA2-low group. **L** Comparison of distant metastasis-free survival (DMFS) between the EPHA2-high group and the EPHA2-low group.

To validate the clinical significance of MFSD4A or EPHA2, we collected samples from 116 patients with NPC from Guilin Medical University Affiliated Hospital diagnosed between November 2012 and March 2013 and conducted immunohistochemical analysis (As for EPHA2, 113 of the 116 patients were included for immunohistochemical analysis, because 3 columns of tissue sections were exhausted). Combined with immunohistochemistry scores and prognostic information for each patient, we classified the patients into the MFSD4A-high group and MFSD4A-low group or the EPHA2-high group and EPHA2-low group. Examples are shown in Fig. 8A, B (MFSD4A) and Fig. 8C, D (EPHA2). The basic clinical information of the patients are shown in Supplementary Table 3. Patients in the MFSD4A-high group had a better overall survival (OS) (Fig. 8E, *P* < 0.001), disease free survival (DFS) (Fig. 8F, *P* = 0.014), and distant metastasis free survival (DMFS) (Fig. 8H, *P* = 0.039), but not loco-regional relapse free survival (LRRFS) (Fig. 8G, *P* = 0.199) compared with those in the MFSD4A-low group. MFSD4A was identified as an independent prognostic factor for OS and DFS in NPC (Supplementary Table 4). More interestingly, EPHA2 could also served as a prognostic marker for OS and DFS (Supplementary Table 5). Patients in the EPHA2-high group had a worse OS, DFS, DMFS, and LRRFS (Fig. 8I–L). Finally, we found that the expression of MFSD4A was negatively correlated with that of EPHA2 by immunohistochemistry assays of serial sections of NPC tissues (Fig. S2 and Supplementary Table 6, *P* < 0.001).

## Discussion

In the present study, we identified the hypermethylation of the promoter region of *MFSD4A* and proved that the expression of *MFSD4A* is reduced and regulated by methylation in NPC. Functionally, inhibition or overexpression of *MFSD4A* promoted or inhibited the proliferation, migration, and invasion of NPC cell lines, respectively, in vitro. In vivo, overexpression of *MFSD4A* resulted in smaller and lighter implanted tumors in mice, as well as a reduction in the number of lung metastases, compared with those in the control group. Mechanistically, MFSD4A targets EPHA2 for ubiquitination and degradation by recruiting RNF149 (a ubiquitin E3 ligase). EPHA2 degradation inhibits downstream PI3K-AKT-ERK1/2 signaling and EMT, ultimately leading to malignant progression of NPC cells. Clinically, immunohistochemistry of samples from 116 patients with NPC showed that the level of MFSD4A correlated with prognosis, and risk stratification was performed to identify high-risk patients. Therefore, MFSD4A is a promising anti-cancer factor, and its role in tumors should not be ignored.

Methylation modification is a common mode of gene expression regulation and is closely associated with tumor progression [20]. Methylation modifications of *HOPX* (encoding HOP homeobox), *RAB37* (encoding Ras-related protein Rab-37), and *SHISA3* (encoding Shisa family member 3) are reported to play an important role in the malignant progression of NPC [21–23]. Our study clarified the correlation between hypermethylation and the expression of *MFSD4A*, which again confirmed the impact of methylation on biological processes, suggesting that the mechanism and significance of methylation regulation tumors is worthy of more detailed study.

MFSD4A was identified as a new antioncogene in the present study and its expression is related to NPC prognosis, which will provide new directions for anti-tumor therapy, and could contribute to predicting prognosis by detecting immunohistochemical indicators in patients with NPC. Previously, we identified methylation levels as a biomarker for prognosis [7] and in this study, we discovered that the expression of methylation-regulated genes can also predict prognosis. It is worth mentioning that immunohistochemistry is a simpler and cheaper method compared with methylation sequencing, and therefore is easier to carry out in clinical practice. In the future, we will verify the prognostic significance of MFSD4A expression in several medical centers. Another finding was that EPHA2 was also an independent prognostic factor for OS and DFS in NPC (Supplementary Table 5). In the previous study [24], EPHA2 was considered as an independent prognostic factor for OS in NPC, but our study further clarified the significance of EPAH2 for DFS.

The MFS family is responsible for the transport of many substances inside and outside cells, and its complex and diverse transport functions maintain normal physiological functions in humans. In recent years, dysregulation of MFS transport proteins has been associated with a variety of tumors. In hepatocellular carcinoma [25], colorectal cancer [26], and neuroblastoma [27], elevated levels of glucose transporter type 1 (GLUT1) were considered to indicate poor prognosis. Exogenous expression of *MFSD2A* in lung cancer cells can induce G1 phase block in vitro, which disrupts cancer cell adhesion and migration, and thus is a novel lung antioncogene [28]. Therefore, our results for MFSD4A, another member of MFS, further reveal the close relationship between MFS and cancer.

EPHA2 was one of the top-ranked proteins binding to MFSD4A in the mass spectrometry analysis (Supplementary Table 2). The prominent role of EPHA2 in cancer attracted our attention [29]. In our study, we discovered that EPHA2 can reverse the suppression of proliferation, migration, and invasion mediated by MFSD4A overexpression in NPC cells, which demonstrated the carcinogenic ability of EPHA2 [24, 30]. *EPHA2* is defined as a downstream gene of *MFSD4A*. By affecting the protein level of EPHA2, MFSD4A achieved an anticancer effect. Further exploration of the molecular mechanism showed that overexpression of *MFSD4A* decreased EPHA2 levels, which was accompanied by changes in the PI3K-AKT-ERK1/2 pathway and EMT. EPHA2 has been reported to be associated with mitogen activated protein kinase (MAPK) pathways. Jing-Hui et al. reported that overexpression of microRNA miR-520e inhibited p38MAPK and ERK1/2 signaling pathways through inhibition of EPHA2, ultimately leading to the suppression of cell proliferation in hepatocellular carcinoma [31]. EPHA2 is able to induce EMT in gastric cancer [32]. Can the decrease of EPHA2 lead to changes to the PI3K-AKT-ERK1/2 pathway and EMT? PI3Ks can be activated by receptors with protein tyrosine kinase activity (Receptor Tyrosine Kinase, RTK) and EPHA2 is a important member of the RTK family [33, 34]. Prema Subbarayal et al have suggested that EPHA2 interacts with the p85 subunit of PI3K to activate the PI3K-AKT signaling pathway [35]. In bladder cancer, Beibei Liu has proved that EPHA2 can promote the growth and metastasis of cancer cells by activating the PI3K/AKT signaling pathway [36]. The results above remind us of the relationship between EPHA2 and signaling pathways. In our study, rescue assays showed reactivation of the PI3K-AKT-ERK1/2 pathway and EMT after *EPHA2* overexpression, confirming that MFSD4A affects the PI3K-AKT-ERK1/2 pathway and EMT by regulating EPHA2, which has not been reported in previous studies. Notably, 740Y-P (Selleck Chemicals, USA, PI3K activator) reversed the phenotype of MFSD4A overexpression or loss of EPHA2 and promoted EMT, suggesting that PI3K-AKT can mediate EMT and promote tumorigenesis, which is consistent with previous study [37].

How does MFSD4A lead to reduced protein levels of EPHA2? Co-IP and mass spectrometry analysis showed that MFSD4A and EPHA2 also bind RNF149. Ubiquitin ligases play an important role in tumors [38] and a previous study suggested that RNF149 binding to wild-type B-Raf proto-oncogene serine/threonine-protein kinase (BRAF) can induce its ubiquitination and proteasomal degradation [17]. We speculated that RNF149 is involved in EPHA2 ubiquitination. This speculation was confirmed using CO-IP assays and immunofluorescence assays, which proved that RNF149 could bind to MFSD4A and EPHA2. Ubiquitination of EPHA2 is a key process in RNF149-mediated degradation of EPHA2. By immunoprecipitation and immunoblot analysis, we found that MFSD4A can mediate the ubiquitination of EPAH2 via RNF149, and when *RNF149* was overexpressed or silenced respectively, EPHA2 ubiquitination was enhanced or down-regulated accordingly. Therefore, we concluded that MFSD4A can mediate the ubiquitination of EPHA2 by recruiting RNF149, followed by EPAH2 degradation.

In this study, we established lung metastasis models using mice to study the effect of MFSD4A on metastasis. Nasopharyngeal carcinoma has the ability to spread to lymph nodes, liver, lung, bone marrow and other organs, among which lung metastasis is very common [39, 40]. Previous investigators have also constructed many metastasis models to simulate metastasis. Cai L et al transplanted nasopharyngeal carcinoma cells into the subhepatic envelope of nude mice to observe the metastasis of liver and lymph nodes [41]. Some researchers planted nasopharyngeal carcinoma cells in the foot-pads of nude mice to observe the metastasis of popliteal lymph nodes [23, 42]. Many other investigators obtained lung metastasis models by tail vein injection [21, 43–47], just as we did. However, the lung metastasis model was not rigorous enough. The key point was that our model ignored the process of tumor entering the bloodstream from the primary site. In fact, before tumor cells can metastasize, they first have to grow at the primary site of the tumor and break through the basement membrane before they can enter the bloodstream and then develop metastasis [48]. Therefore, the ideal mouse model would be to grow NPC cells in the nasopharynx of mice, which was difficult to achieve in practical experiments due to the small nasopharynx of mice. Thus, in the future, we need to explore more superior models for simulating tumor metastasis.

## Conclusion

MFSD4A can recruit RNF149 to degrade EPHA2, thereby inhibiting its downstream PI3K-AKT-ERK1/2 signaling pathway and EMT. This suppresses the proliferation and metastasis of NPC, and thus might provide new ideas and opportunities for targeted therapy of NPC.

## Methods

### Clinical specimens and cell culture

Thirty-four NPC and 15 normal nasopharynx tissues were obtained from Guilin Medical University Affiliated Hospital and used for bisulfite pyrosequencing of the promoter region and *MFSD4A* expression analysis. NPC cell lines (HNE1, HONE1, CNE1, CNE2, 5-8 F, 6-10B, and SUNE1), the NP69 cell line, and HEK293T cells were acquired by email request from Dr. Liu Na (Sun Yat-sen University Cancer Center, China) [21, 47]. Roswell Park Memorial Institute (RPMI)-1640 medium (Invitrogen, Waltham, MA, USA) with 10% fetal bovine serum (FBS, Invitrogen), Keratinocyte serum-free medium (KSFM, Invitrogen), and Dulbecco’s modified Eagle’s medium (DMEM, Invitrogen) supplemented with 10% FBS provided the appropriate components for the growth of NPC, NP69, and HEK293T cells, respectively.

### DNA extraction and bisulfite pyrosequencing

The kits mentioned below were used following the manufacturer’s instructions. NPC cell lines treated with or without 10umol/L 5-aza-2ʹ-deoxycytidine (DAC, Sigma Aldrich, Germany) for 72 h were collected and then an AllPrep RNA/DNA Mini kit (Qiagen, Hilden, Germany) was used to extract DNA from cells and tissues. DNA (1–2 μg) was treated with sodium bisulfite in an EpiTect Bisulfite Kit (Qiagen). Bisulfite pyrosequencing primers for *MFSD4A* was designed using PyroMark Assay Design Software 2.0 (Qiagen) and are displayed in Supplementary Table 1. Sequencing reactions and quantification of methylation levels were achieved using the PyroMark Q96 System (Qiagen).

### Quantitative real-time reverse transcription PCR (qRT-PCR)

The kits mentioned below were used following the manufacturer’s instructions. Total RNA of tissues or cells was extracted using the TRIzol reagent (Invitrogen), and used to synthesize cDNA with a reverse-transcription kit (Promega, Madison, WI, USA). Then, the cDNA was used as a template to perform quantitative real-time PCR reactions using Platinum SYBR Green qPCR SuperMix-UDG reagents (Invitrogen) on an SFX (96) system (Bio-Rad, Hercules, CA, USA). The primers used are shown in Supplementary Table 1.

### Western blotting assays

First, we used Radioimmunoprecipitation assay (RIPA) lysis buffer (Beyotime, Jiangsu, China) to lyse cells to obtain proteins, which were quantified using the Bradford method. The proteins were separated using SDS-polyacrylamide gel electrophoresis (SDS-PAGE, Beyotime) and transferred to polyvinylidene fluoride (PVDF) membranes (Millipore, Billerica, MA, USA). After blocking the membranes for 1 h at room temperature with 5% skim milk, the membranes were incubated with the corresponding primary antibodies at 4 °C overnight. The primary antibodies recognized MFSD4A (Genetex, Irvine, CA, USA), EPHA2 (CST, Danvers, MA, USA), green fluorescent protein (GFP)-tag (Proteintech, USA), HA-tag (Proteintech, Rosemont, IL, USA), Flag-tag (Proteintech), Vimentin (CST), E-cadherin (CST), N-cadherin (CST), Slug (CST), Snail (CST), AKT (CST), phosphorylated (p)-AKT (CST), ERK1/2 (Abcam, Cambridge, MA, USA), p-ERK1/2 (CST), PI3K (Abcam), p-PI3K (CST) and glyceraldehyde-3-phosphate dehydrogenase (GAPDH) (Proteintech). The next day, the corresponding secondary antibodies were added and incubated with the membranes for 1 h at room temperature. Finally, enhanced chemiluminescence was used to observe the results.

### Vector constructure, cell transfection, and lentiviral infection

Small interfering RNAs targeting *MFSD4A*, siMFSD4A (1# and 2#) and siRNF149 (1# and 2#) were obtained from RiboBio (Guangzhou, China) and were used to downregulate the expression of *MFSD4A* and RNF149, respectively. Their sequences are shown in Supplementary Table 1. PCMV3-GFP-vector, pCMV3-GFP-EPHA2, pCMV3-HA-RNF149, and pCMV3-HA-vector were obtained from Sino Biological (Beijing, China). PRK-HA-Ub was provided by Dr. Liu Na (Sun Yat-sen University Cancer Center, China). Phage-puro-6tag-MFSD4A and phage-puro-6tag-vector were constructed following molecular cloning guidelines. Plasmids were transiently transfected into cells using Lipofectamine 3000 (Invitrogen). To generate stable cell lines overexpressing *MFSD4A*, phage-puro-6tag-MFSD4A or phage-puro-6tag-vector (control) were co-transfected with lentivirus packaging plasmids into HEK293T cells. Collected virus was then incubated with SUNE1 and HONE1 cells. After screening NPC cells with puromycin, qRT-PCR and western blotting assays were performed to identify MFSD4A overexpression.

### CCK-8, colony formation, migration, and invasion assays

Cell Counting Kit 8 (CCK-8) assays were used to assess cell proliferation. NPC cells were grown in 96-well plates at 1000 cells per well and monitored continuously for 5 days. Each day, CCK-8 (Dojindo, Kumamoto, Japan) was added to the cell in the wells, and the absorbance values were measured at 450 nm after 2 h. For the colony formation assays, 500 cells were plated in 6-well plates and incubated for 10 days. The colonies formed were fixed with methanol, stained with crystal violet, and counted under a microscope. Migration and invasion assays were performed using Transwell chambers (8 mm pores, Corning Inc., Corning, NY, USA) covered with or without Matrigel (BD Biosciences, San Jose, CA, USA). Serum-free RPMI-1640 medium (200 μl) containing 5 × 10^4^ cells to the upper chamber and 500 μl of RPMI-1640 medium with 20% FBS was added to the lower chamber to induce migration or invasion of cells. Fourteen hours (migration) or 21 h (invasion) later, the upper chambers were collected and the cells were fixed using formaldehyde and stained using crystal violet for observation under the microscope.

### Flow cytometry assays

HONE1 or SUNE1 cells transfected with MFSD4A or co-transfected with MFSD4A and EPHA2 plasmids, then cultured for 36 h, and digested by Trypsin (ThermoFisher, USA), washed by PBS, stained by Annexin V-PE (KeyGEN, China) and finally tested by Flow cytometer.

### Mass spectrometry and co-immunoprecipitation (co-IP) assays

Co-IP was used to demonstrate protein-protein interactions through binding interactions. Mass Spectrometry was applied to identify the specific proteins interacting with MFSD4A. Briefly, we incubated anti-flag, anti-GFP, anti-HA, or anti-IgG antibodies with cell lysates overnight at 4 °C. Then, washed protein A/G magnetic beads (Beyotime) were added to the cell lysate on the mixer to adsorb the immune complexes at room temperature for 1 h. Immune complexes were eluted from the magnetic beads, denatured by boiling, separated using SDS-PAGE, and then subjected to mass spectrometry or western blotting assays.

### Immunofluorescence

5 × 10^4^ cells were seeded per well in 24-well plates. Twelve hours later, the cells were fixed with 4% paraformaldehyde for 15 min, permeabilized with 0.5% Triton X-100 (in phosphate-buffered saline (PBS)) for 15 min, incubated with PBS containing 1% FBS for 30 min at room temperature, and incubated with the corresponding primary antibody overnight at 4 °C. The next day, the specimens were incubated with the corresponding fluorescent secondary antibody for 1 h. After staining the nuclei with 4ʹ,6-diamidino-2-phenylindole (DAPI; Sigma, St. Louis, MO, USA) for 3 min, the specimens were observed under an FV1000 microscope (Olympus, Tokyo, Japan).

### Tumor growth and lung metastasis models in vivo

Twenty BALB/c nude mice were bought from Charles River Laboratories (Beijing, China) to construct tumorigenesis (10 mice) and lung metastasis models (10 mice) in vivo *randomly*. We injected SUNE1 cells (1 × 10^6^) transfected with the empty vector or those stable overexpressing *MFSD4A* into the flanks or caudal vein of mice to construct tumorigenesis or lung metastasis models, respectively. Flank tumors of the mice were obtained to determine the volume and the weight after 30 days, while lungs of mice were dissected to count the number of lung metastases after 60 days.

### Hematoxylin-eosin staining assays

Sections of dissected lung metastasis specimens were dewaxed, stained using hematoxylin and eosin, sealed using neutral gum (Bioworld, Visalia, CA, USA), and finally observed under a microscope.

### Immunohistochemistry assays

Immunohistochemistry assays were applied to detect the expression of MFSD4A or EPHA2. Paraffin sections were first dewaxed and rehydrated using xylene, and then passed through multiple gradient concentrations of ethanol. Endogenous peroxidase was inactivated using 3% H_2_O_2_. Sections were placed in 90 °C water for 6–7 min to repair the antigen, before being blocked with goat serum (Beyotime) and incubated with primary antibodies (anti-MFSD4A or anti-EPHA2, Invitrogen) overnight. The next day, the samples were incubated with biotin-labeled secondary antibody, stained with 3,3ʹ-Diaminobenzidine (DAB) (Agilent Technologies, Santa Clara, CA, USA) and hematoxylin, and finally photographed using a confocal microscopy. Immunohistochemical scoring was performed according to a method detailed in a previous study [49].

### Statistical analyses

Experimental data based on three independent replicate experiments were displayed as the mean ± SD and analyzed statistically using SPSS 24.0 (IBM Corp., Armonk, NY, USA). *T*-tests were used to compare the differences between two groups. The Pearson coefficient was used to evaluate the correlation between the two variables. The chi-square tests were used to compare the composition ratios of the different groups. *P* < 0.05 was regarded as statistically significant.

## Supplementary information

Figure S1

Figure S2

Supplemental Tables

Supplemental Figure legends

Original Data File for WB

Reproducibility checklist

## Data Availability

Data can be obtained from the corresponding author.
