# Supplementary figures and images for: MFSD4A inhibits the malignant progression of nasopharyngeal carcinoma by targeting EPHA2
Source: Cell Death Dis. 2022 Apr 11;13(4):332. doi: 10.1038/s41419-022-04793-x (PMC9001682; doi:10.1038/s41419-022-04793-x)

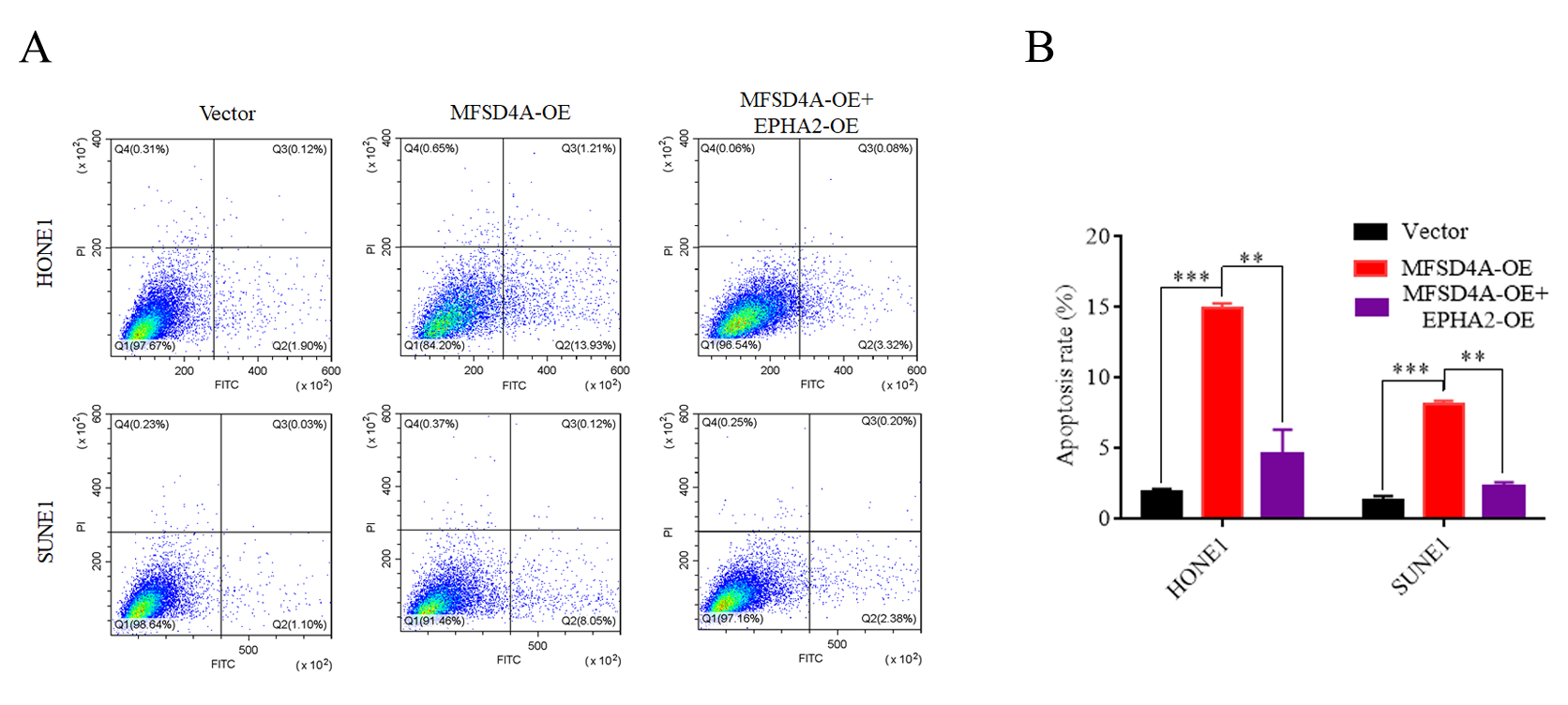

Supplement: Supplementary file 1 — Figure S1 [file 41419_2022_4793_MOESM1_ESM.tif]

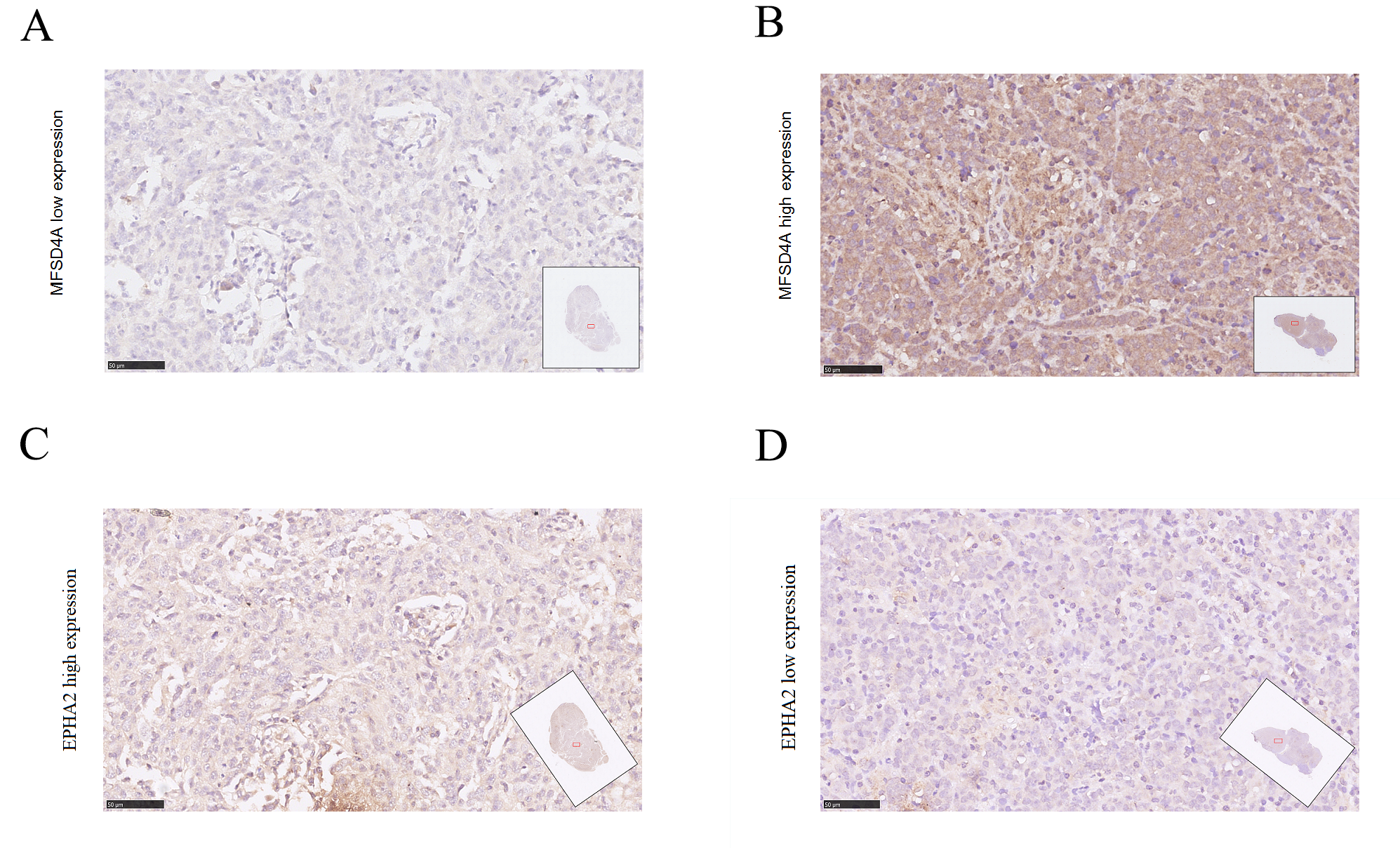

Supplement: Supplementary file 2 — Figure S2 [file 41419_2022_4793_MOESM2_ESM.tif]
