# Supplementary material for: MFSD4A inhibits the malignant progression of nasopharyngeal carcinoma by targeting EPHA2
Source: Cell Death Dis. 2022 Apr 11;13(4):332. doi: 10.1038/s41419-022-04793-x (PMC9001682; doi:10.1038/s41419-022-04793-x)
Supplement: Supplementary file 3 — Supplemental Tables [file 41419_2022_4793_MOESM3_ESM.docx]

Supplementary Table 1. Primers used in this study

| Gene/construct | Sequence (5′ to 3′) |
| --- | --- |
| **BSP*** **PCR primers** |  |
| MFSD4A-F | TGTTTTTTGTTTTGGGGGAAAG |
| MFSD4A-R | ACCAATCCCCCCAACCTC |
| **BSP*** **sequencing primers** |  |
| MFSD4A | ATAAAACTAAAAACCCTCTC |
| **qRT-PCR primers** |  |
| MFSD4A-F | AGACACAGCCTCCTGAGAAGGA |
| MFSD4A-R | CCTCTGAGGTTCTTCCTTTGGC |
| EPHA2-F | TGGCTCACACACCCGTATG |
| EPHA2-R | GTCGCCAGACATCACGTTG |
| GAPDH-F | GAAGGTGAAGG TCGGAGT |
| GAPDH-R | GAAGATGGTGATGGGATTTC |
| **siRNA sequences** |  |
| siMFSD4A#1 | GGATCATGGCCCTCATCAA |
| siMFSD4A#2 | CCTACAACGTCGTCTTCCT |
| siRNF149#1 | CACCGAACATGTCCAATGT |
| siRNF149#2 | GAGTCTAGCTTTACCAGAT |

* BSP, Bisulfite Genomic Sequencing

**Supplemental Table 2: .The top 50 proteins in the MFSD4A pull down assay identified by mass spectrometry analysis.**

| **Protein** | **Score** |
| --- | --- |
| SLC25A4 | 1567 |
| EPHA2 | 1543 |
| MFSD4A | 1467 |
| RPN1 | 960 |
| HSPA1A | 926 |
| SLC25A3 | 677 |
| HSD17B12 | 657 |
| SAMM50 | 644 |
| SLC25A13 | 543 |
| SLC3A2 | 481 |
| TUFM | 474 |
| SLC25A12 | 460 |
| SRPRB | 447 |
| HSPA9 | 446 |
| CLTC | 445 |
| RPS3 | 416 |
| FDFT1 | 379 |
| HNRNPH1 | 356 |
| ATP5F1B | 339 |
| RPS27A | 335 |
| ATP2A2 | 335 |
| PTPRF | 325 |
| RNF149 | 314 |
| UQCRC2 | 301 |
| EEF1A1P5 | 295 |
| SLC25A1 | 292 |
| GLUD1 | 275 |
| DHCR7 | 266 |
| VDAC2 | 264 |
| XRCC6 | 261 |
| ALB | 259 |
| PTPN1 | 257 |
| HLA-B | 250 |
| APOOL | 250 |
| SLC25A22 | 246 |
| LMNA | 244 |
| MCU | 243 |
| KRT8 | 242 |
| SQSTM1 | 238 |
| TAP1 | 231 |
| CAVIN1 | 231 |
| ACTBL2 | 231 |
| DDOST | 229 |
| ATP5F1C | 228 |
| RPS4X | 227 |
| KRT17 | 225 |
| PHB | 216 |
| VAPA | 214 |
| PI4K2A | 210 |
| CHCHD6 | 208 |

**Supplemental Table 3. Relationship between MFSD4A expression and clinicopathological features of patients with NPC (N = 116)**

| **Characteristic** | **MFSD4A-high group (N, %)** | **MFSD4A-low group (N, %)** | ***p*-value** |
| --- | --- | --- | --- |
| **Age** |  |  |  |
| ≤ 48 years | 36 (53.73) | 24 (48.98) | 0.613 |
| > 48 years | 31 (46.37) | 25 (51.02) |  |
| **Sex** |  |  |  |
| Male | 42 (62.69) | 41 (83.67) | 0.013 |
| Female | 25 (37.31) | 8 (16.33) |  |
| **Clinical T stage** |  |  |  |
| T1 | 11 (16.42) | 4 (8.16) | 0.165 |
| T2 | 17 (25.37) | 7 (14.29) |  |
| T3 | 13 (19.40) | 15 (30.61) |  |
| T4 | 26 (38.81) | 23 (46.94) |  |
| **Clinical N stage** |  |  |  |
| N0 | 2 (2.98) | 1 (2.04) | 0.047 |
| N1 | 6 (8.96) | 8 (16.33) |  |
| N2 | 55 (82.09) | 30 (61.22) |  |
| N3 | 4 (5.97) | 10 (20.41) |  |
| **TNM stage** |  |  |  |
| II | 4 (5.97) | 5 (10.20) | 0.064 |
| III | 34 (50.75) | 17 (34.70) |  |
| IVa | 25 (37.31) | 17 (34.70) |  |
| IVb | 4 (5.97) | 10 (20.40) |  |
| **Induction chemotherapy** |  |  | 0.837 |
| Yes | 30 (44.78) | 21 (42.86) |  |
| No | 37 (55.22) | 28 (57.14) |  |
| **Concurrent chemotherapy** |  |  |  |
| Yes | 63 (94.03) | 43 (87.76) | 0.234 |
| No | 4 (5.97) | 6 (12.24) |  |
| **Adjuvant chemotherapy** |  |  |  |
| Yes | 10 (14.93) | 5 (10.20) | 0.454 |
| No | 57 (85.07) | 44 (89.80) |  |

**The *p-*value was determined using χ^2^ tests.**

**Supplemental Table 4: Cox regression analysis of clinical variables contributing to overall survival, disease-free survival, locoregional relapse free survival, and distant metastasis-free survival in patients with NPC (N = 116)**

| **Variables** | **Univariate analysis** | | | **Multivariate analysis** | | |
| --- | --- | --- | --- | --- | --- | --- |
|  | **Hazard ratio** | **95% CI** | ***p*-value** | **Hazard ratio** | **95% CI** | ***p*-value** |
| Overall survival |  |  |  |  |  |  |
| Age | 1.021 | 0.987–1.056 | 0.225 |  |  |  |
| Sex (male *vs*. female) | 0.377 | 0.145–0.976 | 0.044 | 0.381 | 0.143–1.015 | 0.054 |
| TNM stage | 2.258 | 1.445–3.529 | < 0.001 | 2.268 | 1.446–3.556 | < 0.001 |
| Induction chemotherapy (No *vs*. Yes) | 1.096 | 0.552–2.175 | 0.793 |  |  |  |
| Concurrent chemotherapy (No *vs*. Yes) | 0.594 | 0.209–1.690 | 0.329 |  |  |  |
| Adjuvant chemotherapy (No *vs*. Yes) | 1.358 | 0.524–3.518 | 0.528 |  |  |  |
| MFSD4A groups (Low *vs*.High) | 0.231 | 0.110–0.486 | < 0.001 | 0.288 | 0.134–0.619 | 0.001 |
| Disease free survival |  |  |  |  |  |  |
| Age | 0.974 | 0.942–1.008 | 0.132 |  |  |  |
| Sex (male *vs*. female) | 0.972 | 0.450–2.100 | 0.942 |  |  |  |
| TNM stage | 1.872 | 1.214–2.886 | 0.005 | 1.720 | 1.122–2.638 | 0.013 |
| Induction chemotherapy (No *vs*. Yes) | 0.738 | 0.361–1.512 | 0.407 |  |  |  |
| Concurrent chemotherapy (No *vs*. Yes) | 3.115 | 0.425–22.821 | 0.263 |  |  |  |
| Adjuvant chemotherapy (No *vs*. Yes) | 1.005 | 0.353–2.866 | 0.992 |  |  |  |
| MFSD4A groups (Low *vs*. High) | 0.412 | 0.204–0.836 | 0.014 | 0.466 | 0.228–0.956 | 0.037 |
| Locoregional relapse free survival |  |  |  |  |  |  |
| Age | 0.963 | 0.919–1.008 | 0.106 |  |  |  |
| Sex (male *vs*. female) | 1.284 | 0.482–3.422 | 0.607 |  |  |  |
| TNM stage | 1.987 | 1.119–3.528 | 0.019 | 1.987 | 1.119–3.528 | 0.019 |
| Induction chemotherapy (No *vs*. Yes) | 0.335 | 0.110–1.019 | 0.054 |  |  |  |
| Concurrent chemotherapy (No *vs*. Yes) | 1.540 | 0.205–11.573 | 0.675 |  |  |  |
| Adjuvant chemotherapy (No *vs*. Yes) | 0.039 | 0.000–12.909 | 0.274 |  |  |  |
| MFSD4A groups (Low *vs*. High) | 0.543 | 0.214–1.377 | 0.199 |  |  |  |
| Distant metastasis free survival |  |  |  |  |  |  |
| Age | 0.983 | 0.943–1.025 | 0.425 |  |  |  |
| Sex (male *vs*. female) | 0.572 | 0.192–1.699 | 0.314 |  |  |  |
| TNM stage | 1.778 | 1.039–3.042 | 0.036 | 1.612 | 0.948–2.742 | 0.078 |
| Induction chemotherapy (No *vs*. Yes) | 0.949 | 0.400–2.252 | 0.906 |  |  |  |
| Concurrent chemotherapy (No *vs*. Yes) | 1.961 | 0.263–14.614 | 0.511 |  |  |  |
| Adjuvant chemotherapy (No *vs*. Yes) | 1.722 | 0.579–5.119 | 0.328 |  |  |  |
| MFSD4A groups (Low *vs*. High) | 0.396 | 0.164–0.955 | 0.039 | 0.448 | 0.183–1.099 | 0.079 |

**The *p-*values were calculated using univariate or multivariate Cox regression analysis. TNM, tumor-node-metastasis; CI, confidence interval.**

**Supplemental Table 5: Cox regression analysis of clinical variables contributing to overall survival, disease-free survival, locoregional relapse free survival, and distant metastasis-free survival in patients with NPC (N = 113)**

| **Variables** | **Univariate analysis** | | | **Multivariate analysis** | | |
| --- | --- | --- | --- | --- | --- | --- |
|  | **Hazard ratio** | **95% CI** | ***p*-value** | **Hazard ratio** | **95% CI** | ***p*-value** |
| Overall survival |  |  |  |  |  |  |
| Age | 1.018 | 0.984 | 1.053 |  |  |  |
| Sex (male *vs*. female) | 0.395 | 0.152-1.026 | 0.057 |  |  |  |
| TNM stage | 2.214 | 1.403-3.494 | 0.001 | 1.454 | 0.898-2.354 | 0.128 |
| Induction chemotherapy (No *vs*. Yes) | 0.993 | 0.494-1.997 | 0.985 |  |  |  |
| Concurrent chemotherapy (No *vs*. Yes) | 0.780 | 0.238-2.561 | 0.682 |  |  |  |
| Adjuvant chemotherapy (No *vs*. Yes) | 1.087 | 0.381-3.098 | 0.876 |  |  |  |
| EPHA2 groups (Low *vs*.High) | 13.307 | 5.104-34.691 | < 0.001 | 11.129 | 4.154-29.815 | < 0.001 |
| Disease free survival |  |  |  |  |  |  |
| Age | 0.977 | 0.944-1.010 | 0.169 |  |  |  |
| Sex (male *vs*. female) | 0.977 | 0.452-2.111 | 0.952 |  |  |  |
| TNM stage | 1.841 | 1.193-2.840 | 0.006 | 1.333 | 0.839-2.118 | 0.224 |
| Induction chemotherapy (No *vs*. Yes) | 0.728 | 0.356-1.491 | 0.386 |  |  |  |
| Concurrent chemotherapy (No *vs*. Yes) | 2.829 | 0.386-20.726 | 0.306 |  |  |  |
| Adjuvant chemotherapy (No *vs*. Yes) | 1.070 | 0.375-3.052 | 0.899 |  |  |  |
| EPHA2 groups (Low *vs*. High) | 4.674 | 2.209-9.889 | < 0.001 | 3.910 | 1.749-8.740 | 0.001 |
| Locoregional relapse free survival |  |  |  |  |  |  |
| Age | 0.965 | 0.921-1.010 | 0.129 |  |  |  |
| Sex (male *vs*. female) | 1.290 | 0.484-3.438 | 0.610 |  |  |  |
| TNM stage | 1.955 | 1.100-3.474 | 0.022 | 1.527 | 0.834-2.796 | 0.170 |
| Induction chemotherapy (No *vs*. Yes) | 0.330 | 0.109-1.006 | 0.051 |  |  |  |
| Concurrent chemotherapy (No *vs*. Yes) | 1.390 | 0.185-10.448 | 0.749 |  |  |  |
| Adjuvant chemotherapy (No *vs*. Yes) | 0.040 | 0.000-14.578 | 0.285 |  |  |  |
| EPHA2 groups (Low *vs*. High) | 3.670 | 1.377-9.784 | 0.009 | 2.834 | 0.988-8.131 | 0.053 |
| Distant metastasis free survival |  |  |  |  |  |  |
| Age | 0.985 | 0.945-1.027 | 0.487 |  |  |  |
| Sex (male *vs*. female) | 0.575 | 0.193-1.709 | 0.319 |  |  |  |
| TNM stage | 1.748 | 1.020-2.995 | 0.042 | 1.238 | 0.690-2.221 | 0.475 |
| Induction chemotherapy (No *vs*. Yes) | 0.937 | 0.395-2.224 | 0.883 |  |  |  |
| Concurrent chemotherapy (No *vs*. Yes) | 1.791 | 0.240-13.345 | 0.570 |  |  |  |
| Adjuvant chemotherapy (No *vs*. Yes) | 1.829 | 0.615-5.438 | 0.278 |  |  |  |
| EPHA2 groups (Low *vs*. High) | 4.829 | 1.872-12.457 | 0.001 | 4.219 | 1.524-11.678 | 0.006 |

**The *p-*values were calculated using univariate or multivariate Cox regression analysis. TNM, tumor-node-metastasis; CI, confidence interval.**

**Supplemental Table 6: Correlation analysis of MFSD4A expression and EPHA2 expression.**

|  |  | MFSD4A expression | EPHA2 expression |
| --- | --- | --- | --- |
| **MFSD4A expression** | Pearson Correlation | 1 | -0.426^**^ |
|  | Sig. (2-tailed) |  | 0.000 |
|  | N | 113 | 113 |
| **EPHA2 expression** | Pearson Correlation | -0.426^**^ | 1 |
|  | Sig. (2-tailed) | 0.000 |  |
|  | N | 113 | 113 |

****. Correlation is significant at the 0.01 leval (2-tailed)**
