# Supplementary material for: MFSD4A inhibits the malignant progression of nasopharyngeal carcinoma by targeting EPHA2
Source: Cell Death Dis. 2022 Apr 11;13(4):332. doi: 10.1038/s41419-022-04793-x (PMC9001682; doi:10.1038/s41419-022-04793-x)
Supplement: Supplementary file 4 — Supplemental Figure legends [file 41419_2022_4793_MOESM4_ESM.doc]

Figure S1 MFSD4A induced apoptosis of NPC cells by inhibiting EPHA2

1. Apoptosis detection of the HONE1 or SUNE1 cells transfected with vector (left) or MFSD4A (middle) or co-transfected with MFSD4A and EPHA2 plasmids (right) by Flow cytometry assays. (B) Comparison of apoptosis rates of the HONE1 or SUNE1 cells transfected with vector, transfected with MFSD4A, co-transfected with MFSD4A and EPHA2 plasmids.

Figure S2 Immunohistochemistry assays of serial sections of NPC tissues for MFSD4A and EPHA2.

(A-B) The immunohistochemical staining of a sample from a patient in the MFSD4A-low group (A) and from a patient in the MFSD4A-high group (B). (C-D) The immunohistochemical staining of a sample from a patient in the EPHA2-low group (C) and from a patient in the EPHA2-high group (D). A and C were serial sections of NPC tissues. B and D were serial sections of NPC tissues.
