# Supplementary material for: MFSD4A inhibits the malignant progression of nasopharyngeal carcinoma by targeting EPHA2
Source: Cell Death Dis. 2022 Apr 11;13(4):332. doi: 10.1038/s41419-022-04793-x (PMC9001682; doi:10.1038/s41419-022-04793-x)
Supplement: Supplementary file 5 — Original Data File for WB [file 41419_2022_4793_MOESM5_ESM.doc]

Figure 2C

Origin Figure


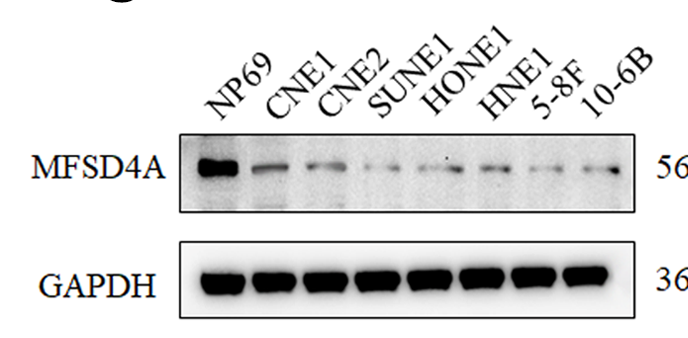


MFSD4A


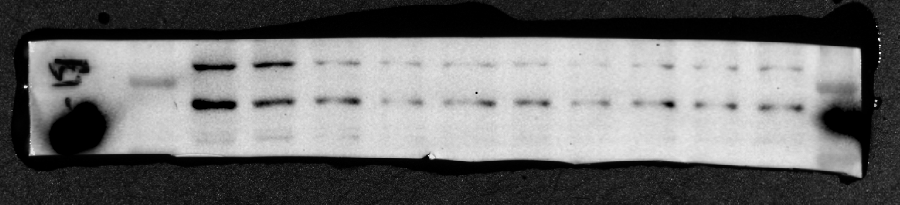


GAPDH


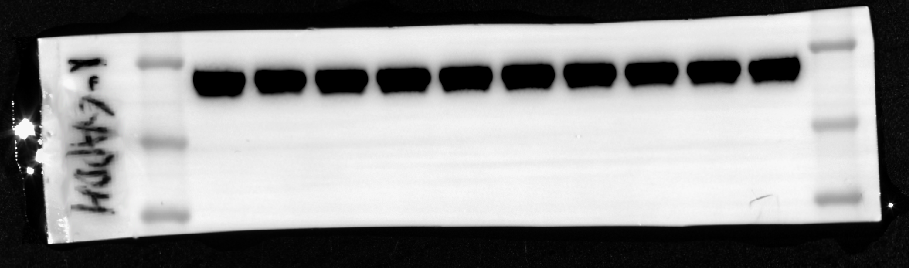


Figure 2D

Origin Figure


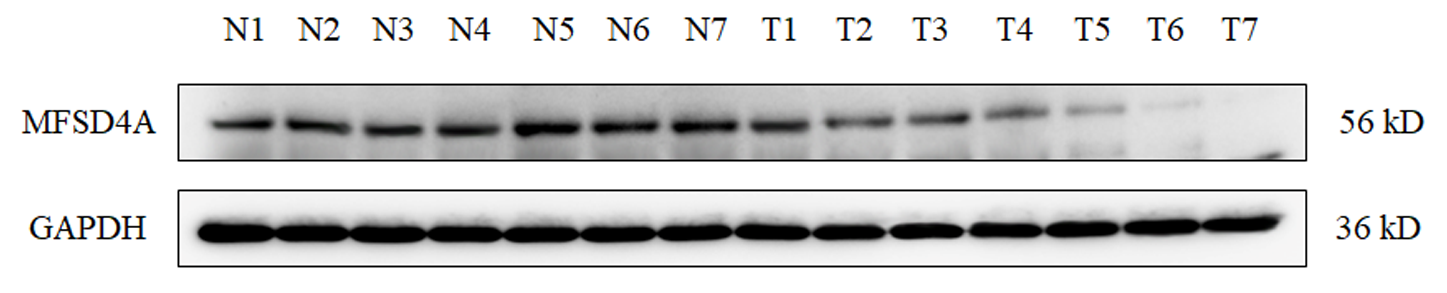


GAPDH


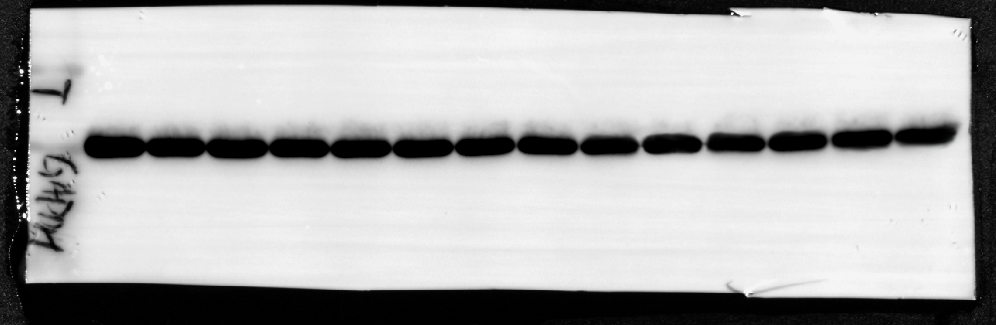


MFSD4A


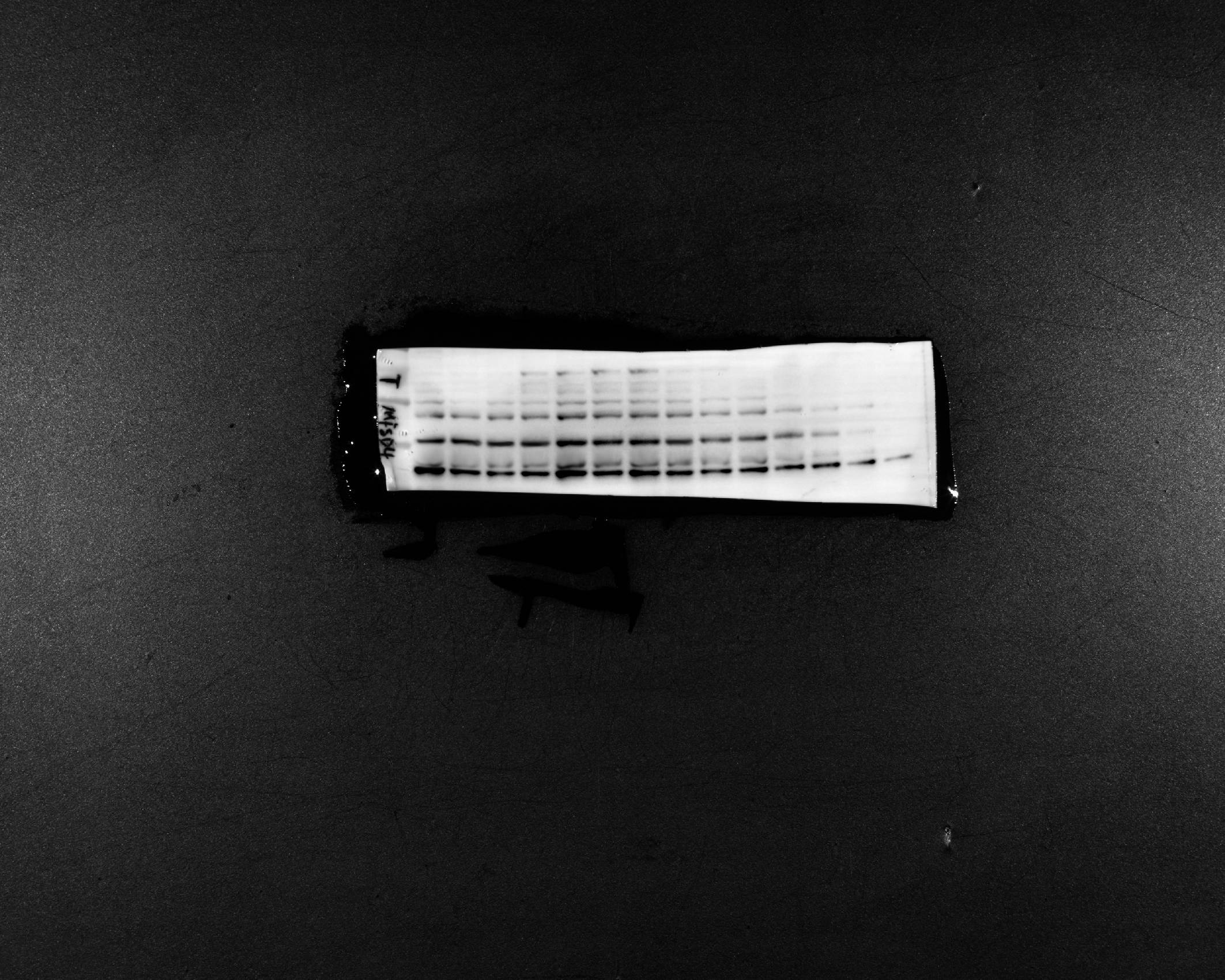


Figure 3B

Origin Figure


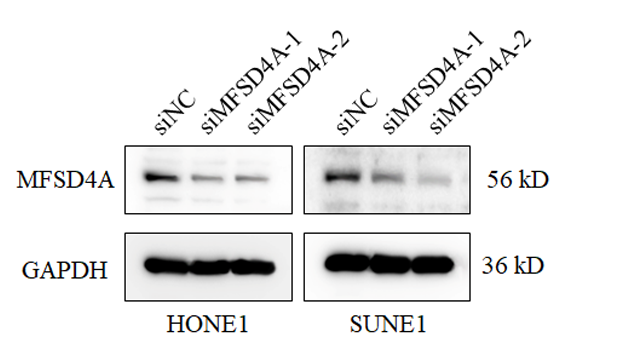


GAPDH

HONE1 SUNE1


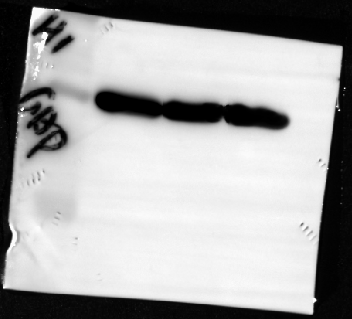

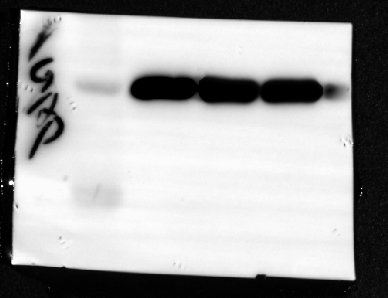


MFSD4A

HONE1 SUNE1


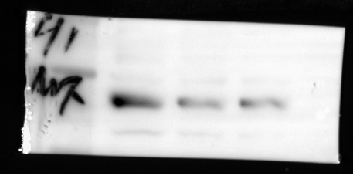

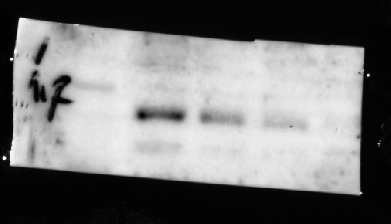


Figure 3D

Origin Figure


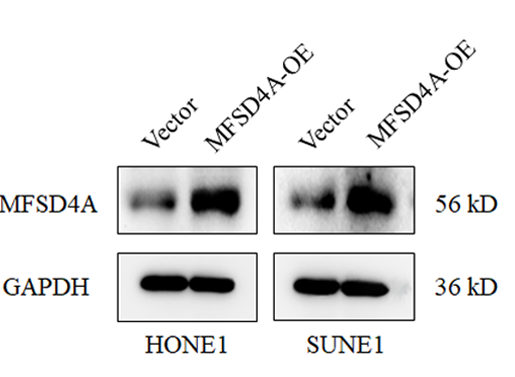


GAPDH

HONE1 SUNE1


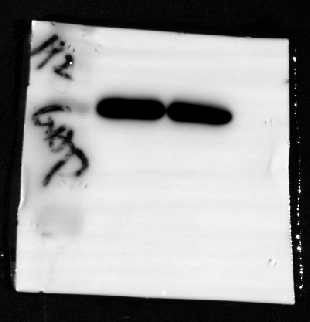

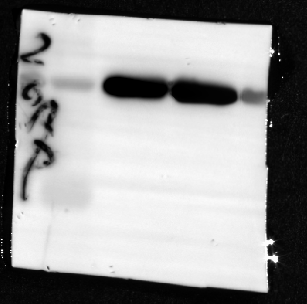


MFSD4A

HONE1 SUNE1


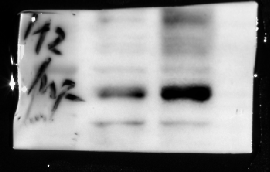

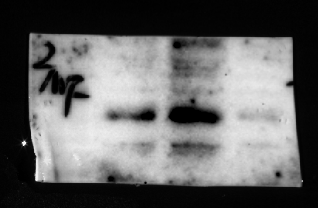


Figure 3M

Origin Figure


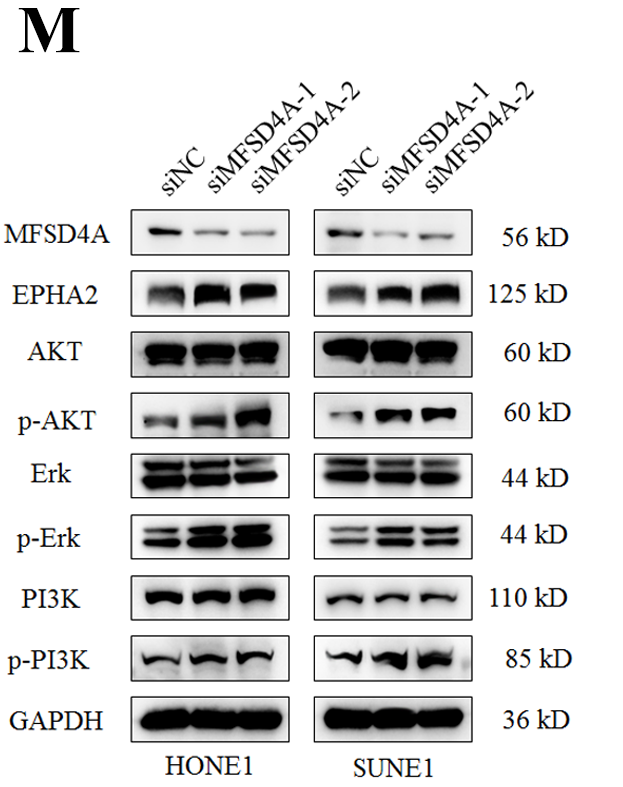


MFSD4A

HONE1 SUNE1


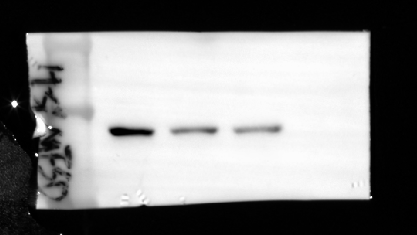

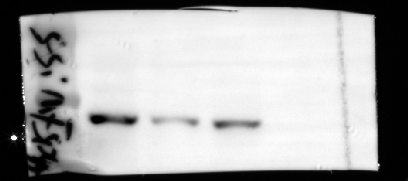


EPHA2

HONE1 SUNE1


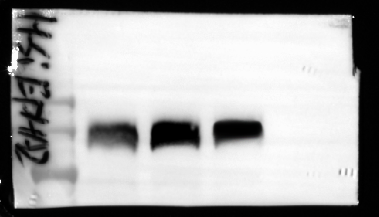

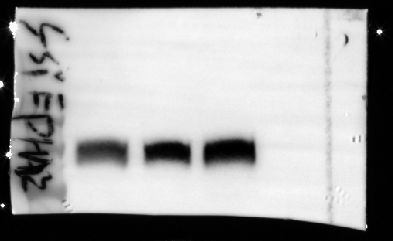


AKT

HONE1 SUNE1


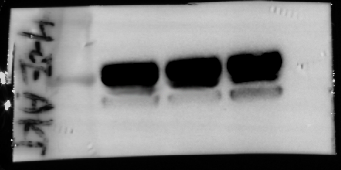

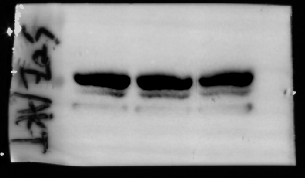


1. AKT

HONE1 SUNE1


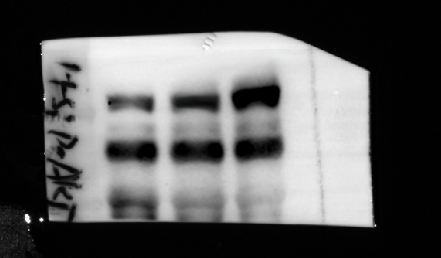

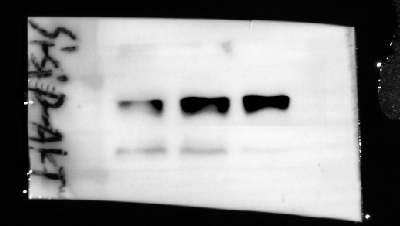


ERK

HONE1 SUNE1


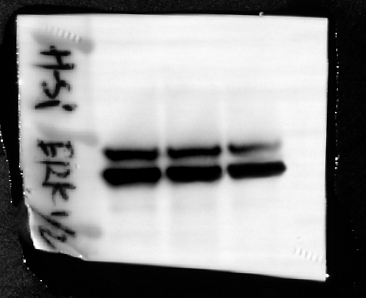

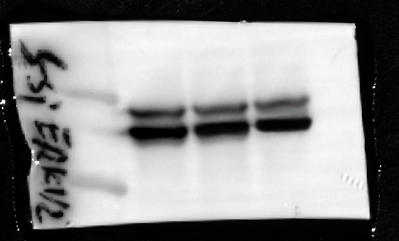


P-ERK

HONE1 SUNE1


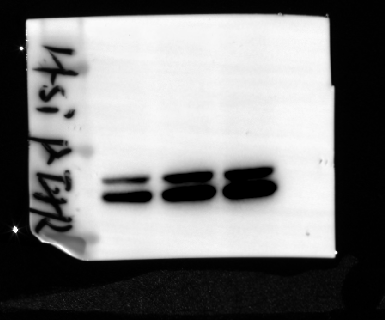

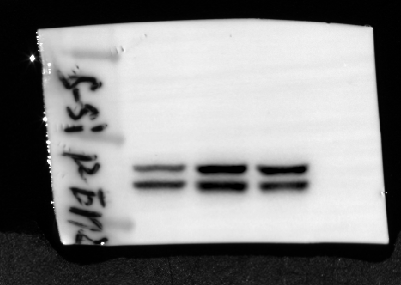


PI3K

HONE1 SUNE1


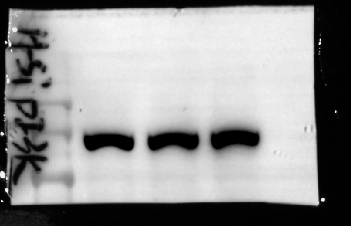

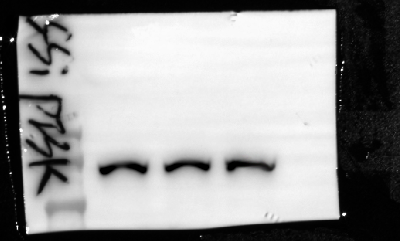


P-PI3K

HONE1 SUNE1


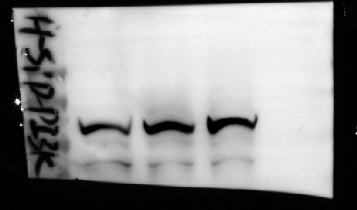

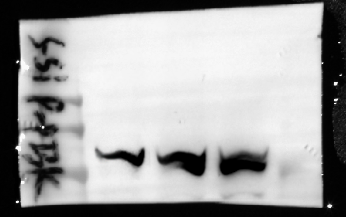


GAPDH

HONE1 SUNE1


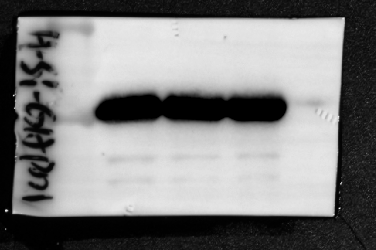

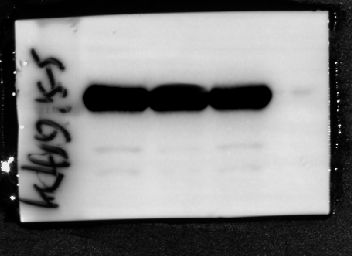


Figure 3N

Origin Figure


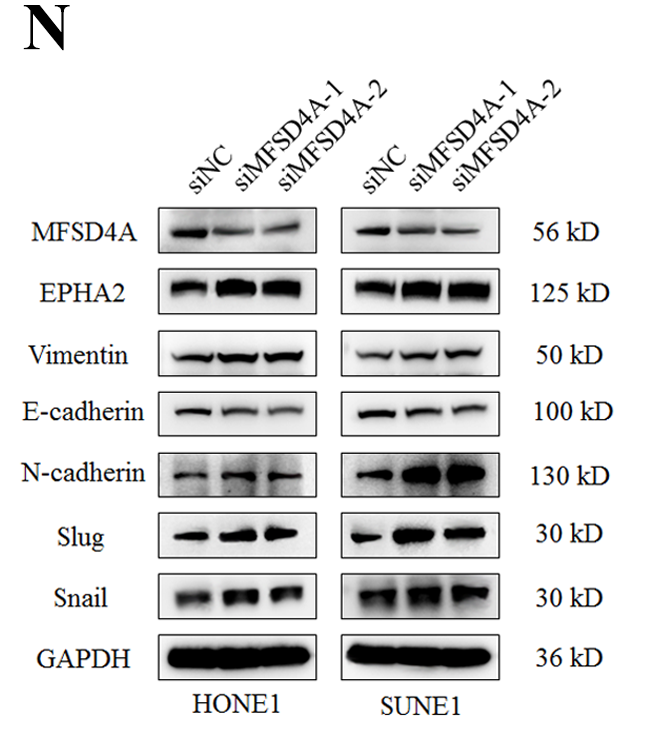


MFSD4A

HONE1 SUNE1


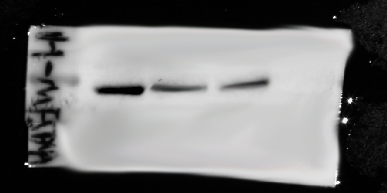

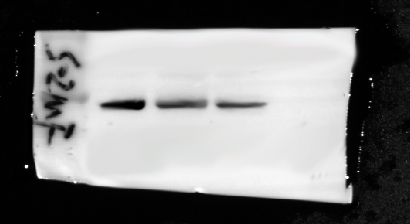


EPHA2

HONE1 SUNE1


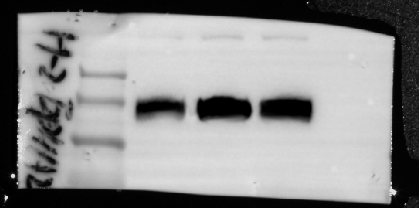

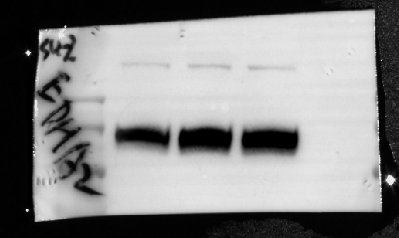


Vitmentin

HONE1 SUNE1


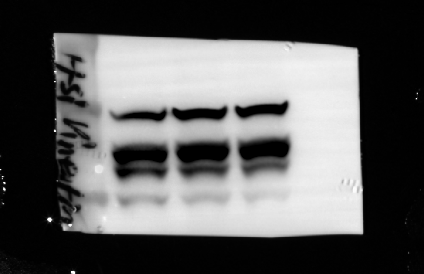

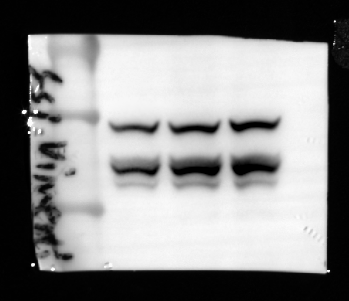


1. Cadherin

HONE1 SUNE1


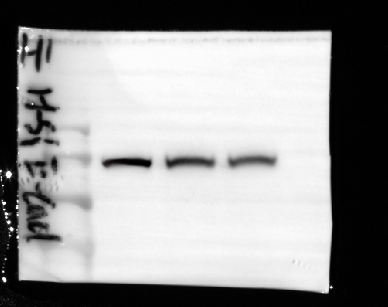

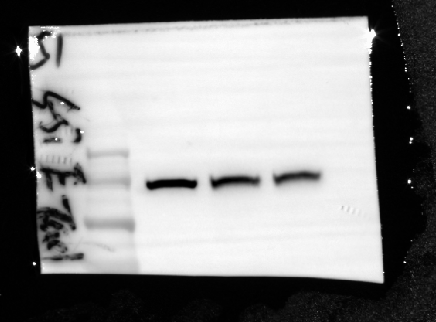


N-cadherin

HONE1 SUNE1


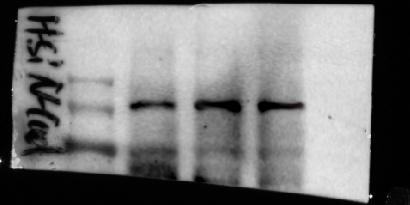

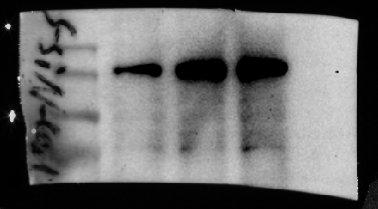


Slug

HONE1 SUNE1


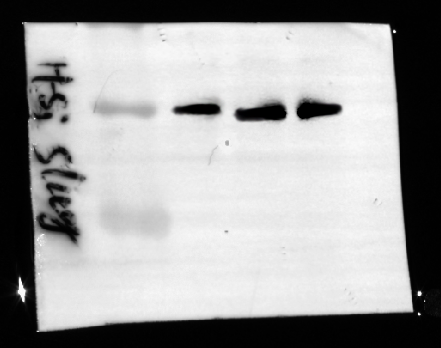

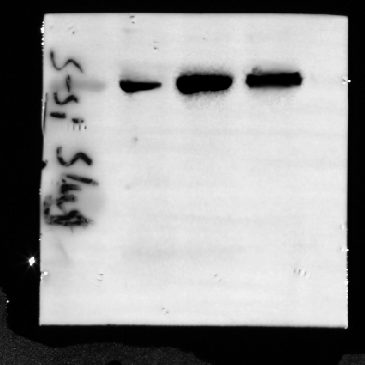


Snail

HONE1 SUNE1


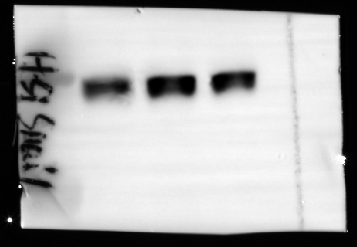

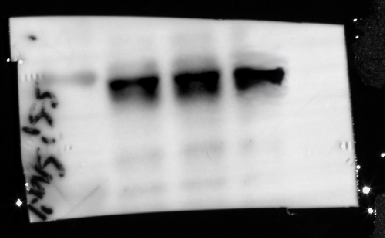


GAPDH

HONE1 SUNE


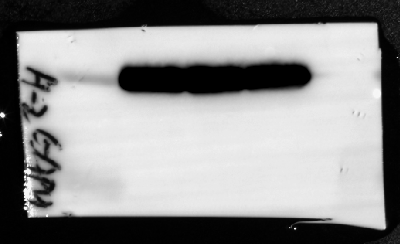

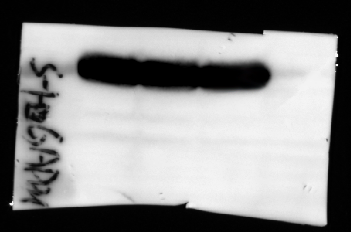


Figure 4A

Origin Figure


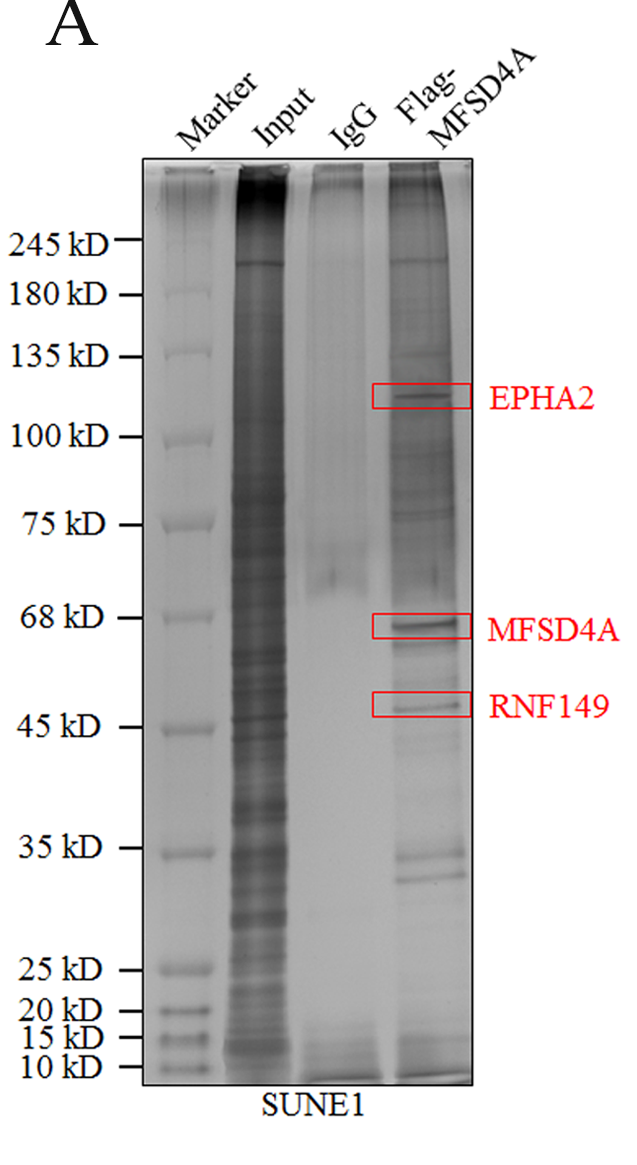


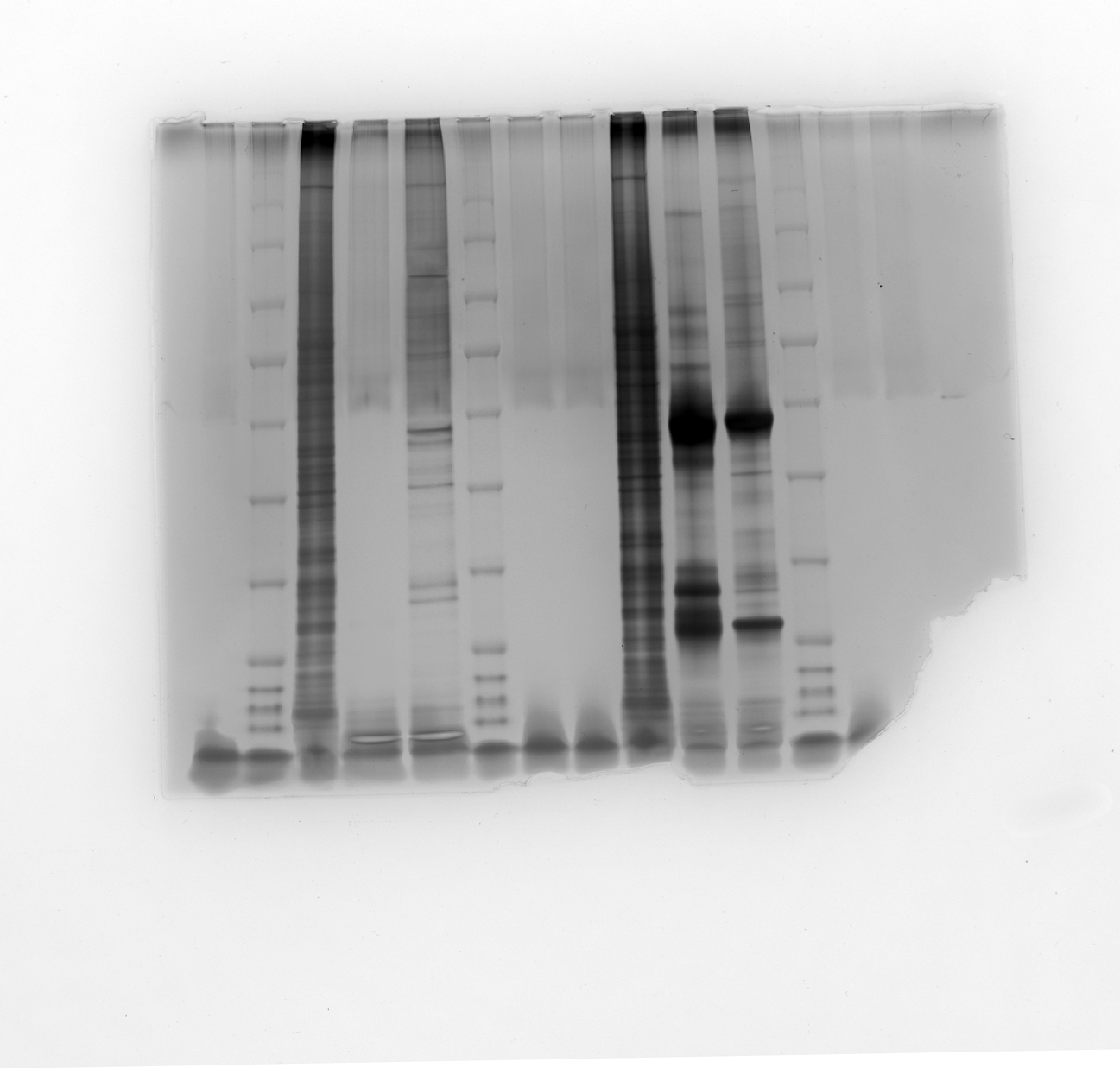


Figure 4B

Origin Figure


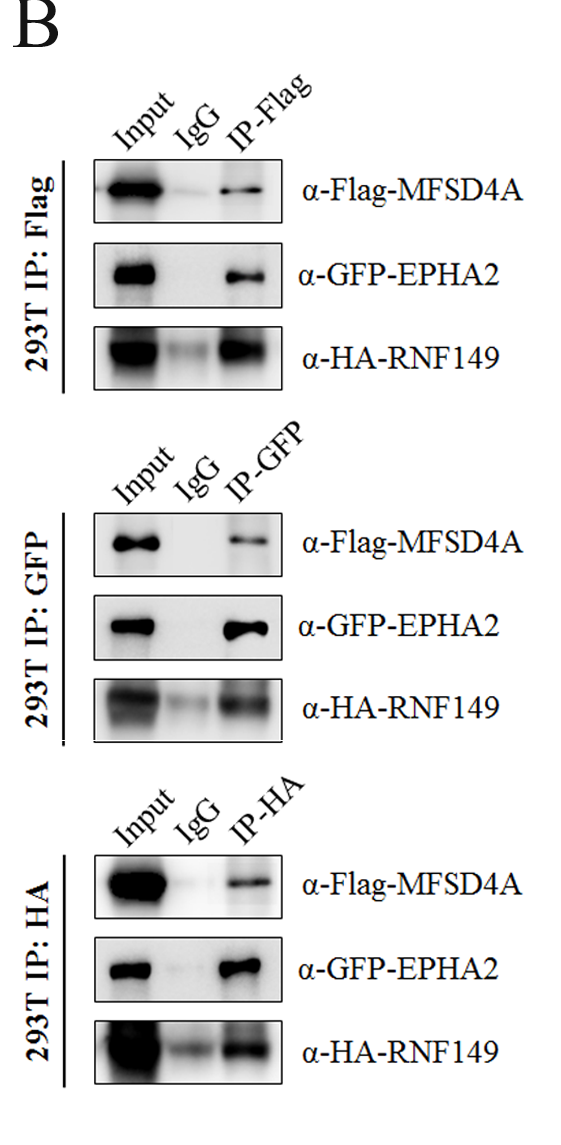


IP:Flag

WB:flag-MFSD4A WB:GFP-EPHA2 WB: HA-RNF149


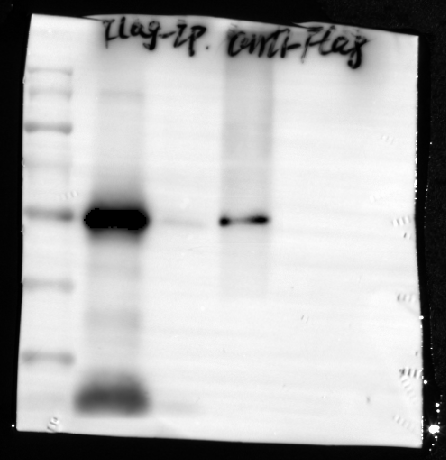

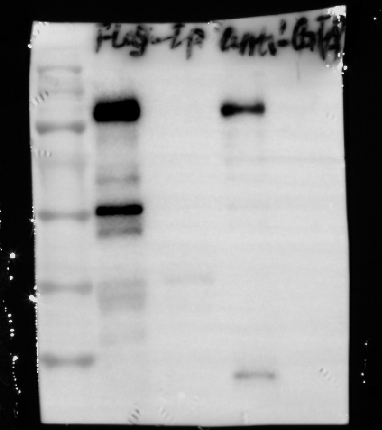

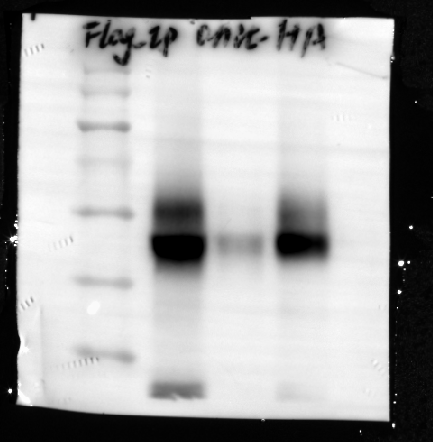


IP:GFP

WB:flag-MFSD4A WB:GFP-EPHA2 WB: HA-RNF149


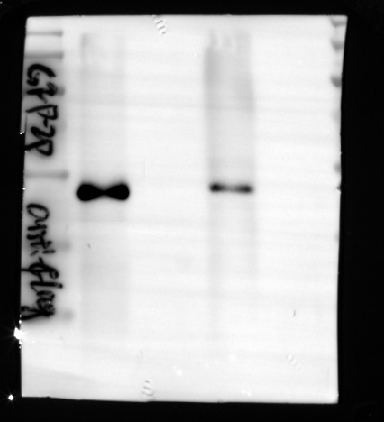

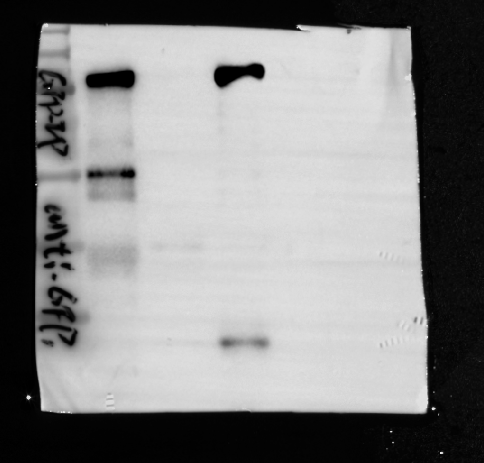

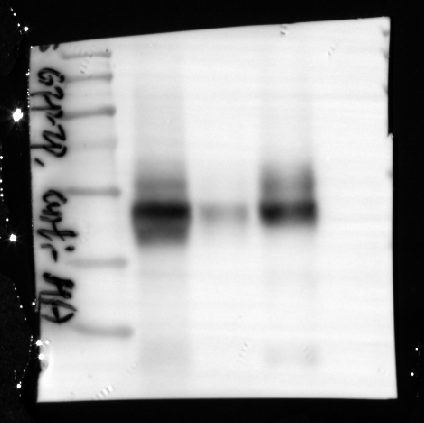


IP:HA

WB:flag-MFSD4A WB:GFP-EPHA2 WB: HA-RNF149


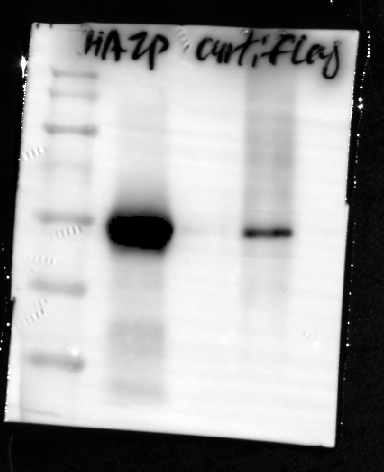

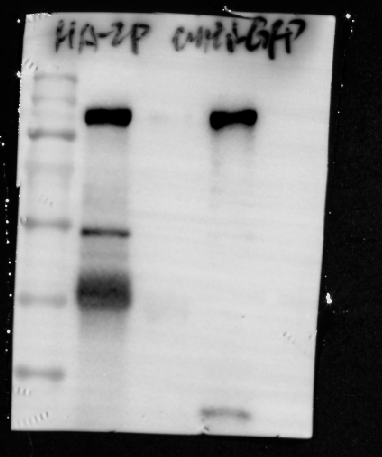

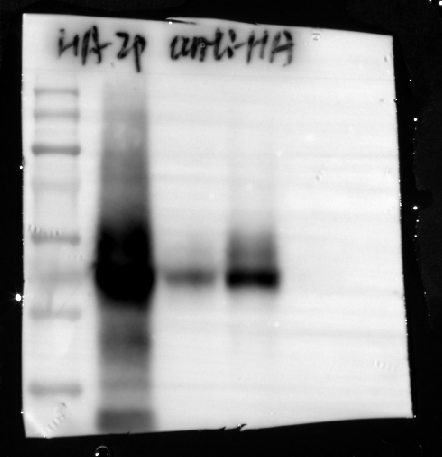


Figure 5C

Origin Figure


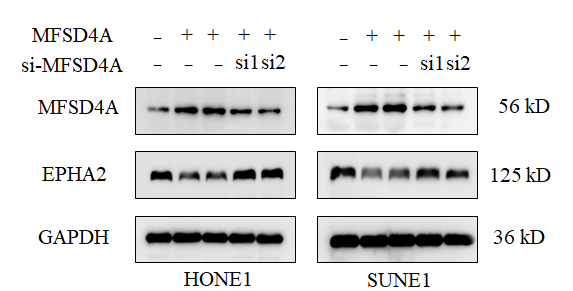


MFSD4A

HONE1 SUNE1


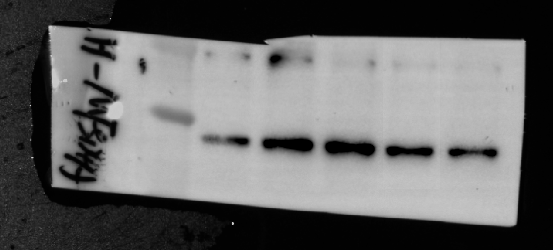

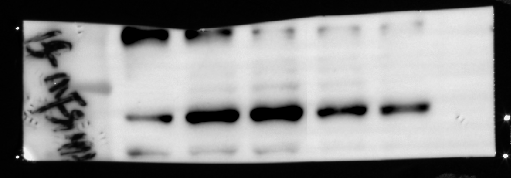


EPHA2

HONE1 SUNE1


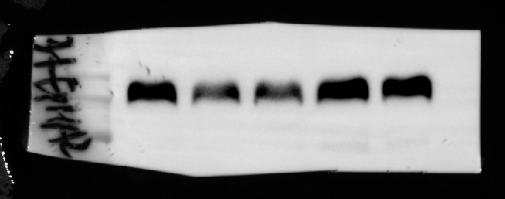

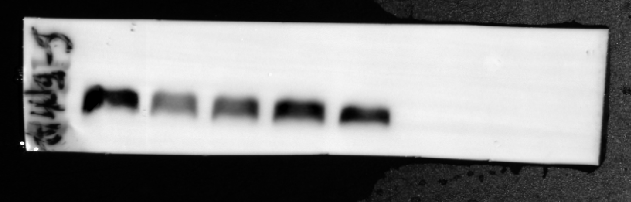


GAPDH

HONE1 SUNE1


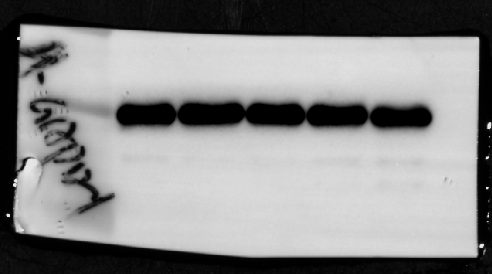

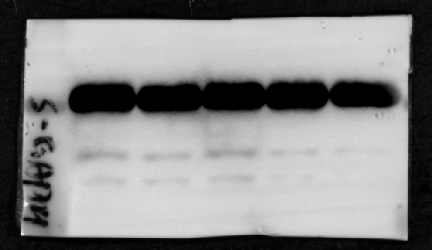


Figure 5D

Origin Figure


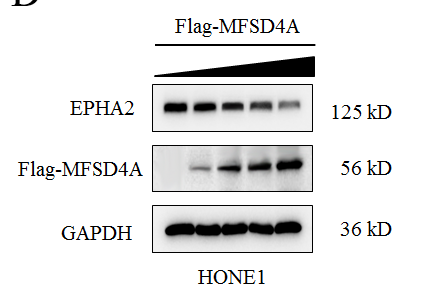


EPHA2 Flag-MFSD4A


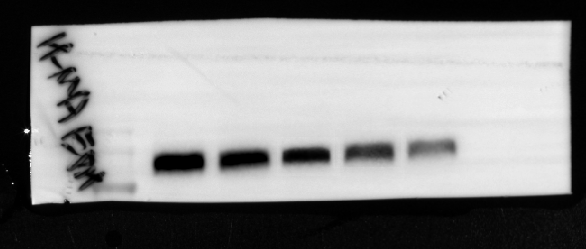

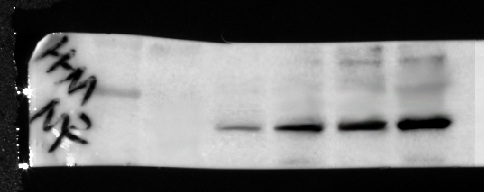


GAPDH


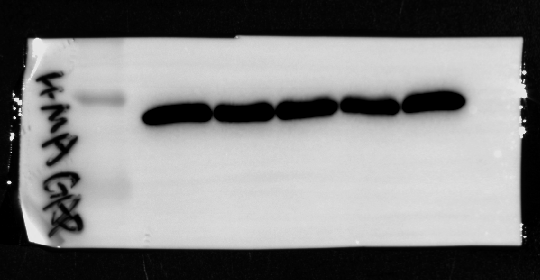


Figure 5E

Origin Figure


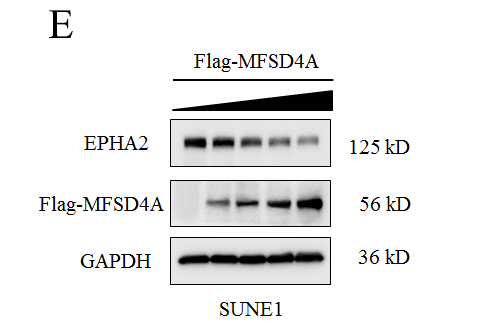


EPHA2 Flag-MFSD4A


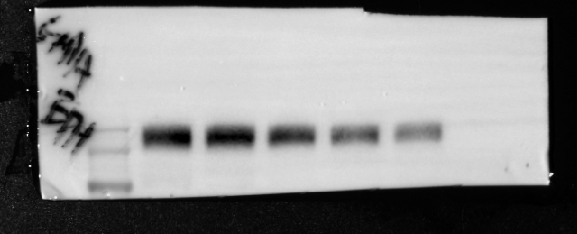

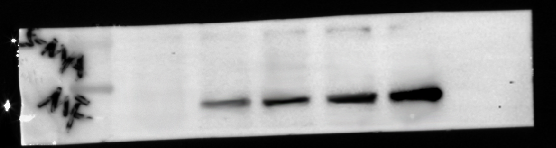


GAPDH


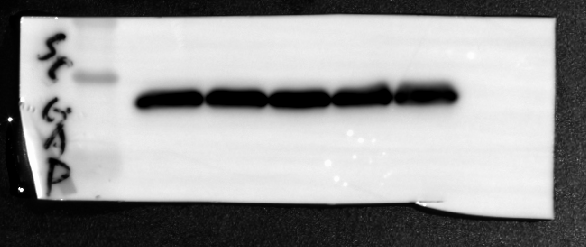


Figure 5F

Origin Figure


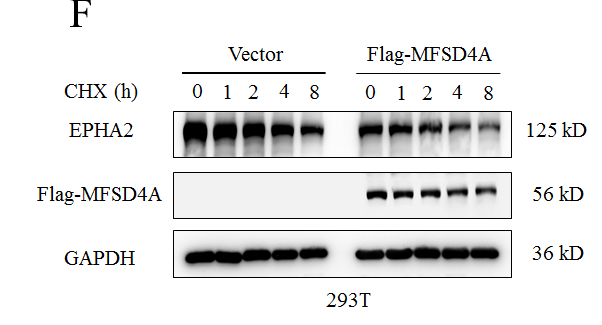


EPHA2

Vector Flag-MFSD4A


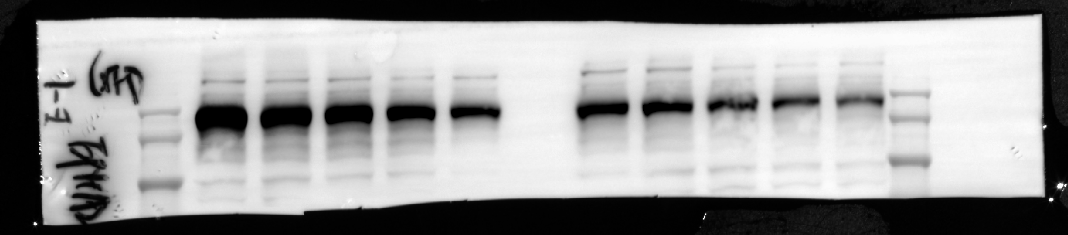


Flag-MFSD4A

Vector Flag-MFSD4A


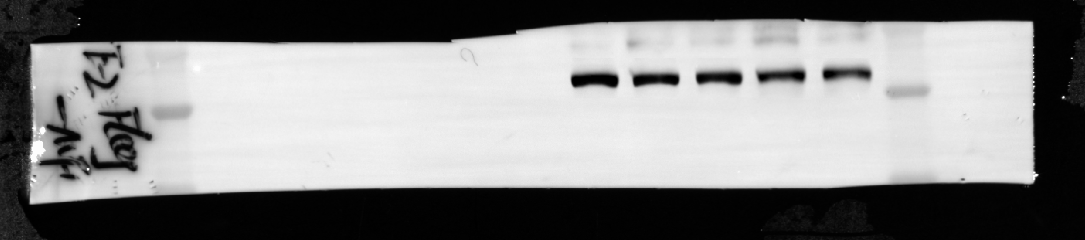


GAPDH

Vector Flag-MFSD4A


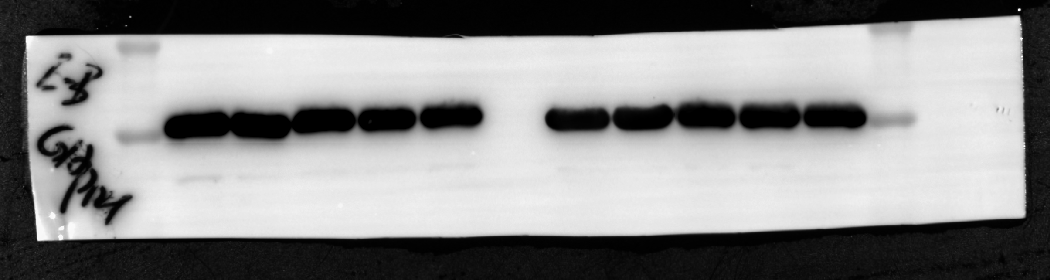


Figure 5G

Origin Figure


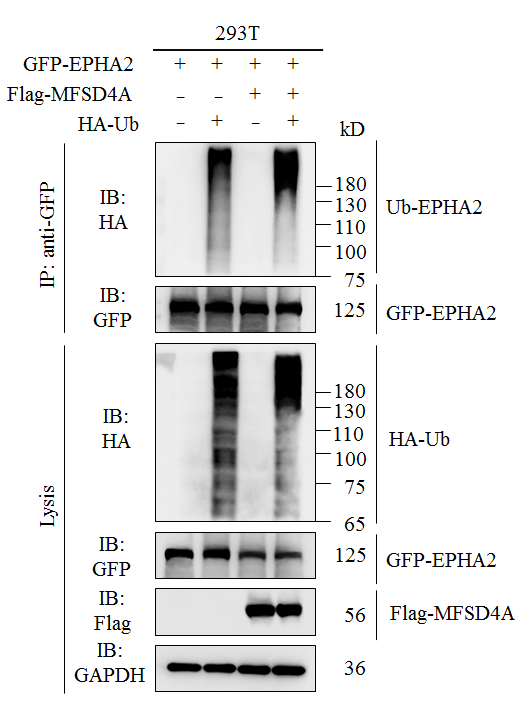


IP:GFP

WB:UB-EPHA2 WB:GFP-EPHA2 Lysis WB:HA-Ub


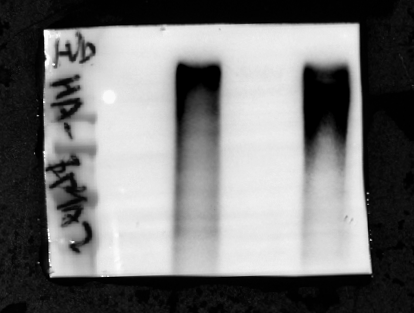

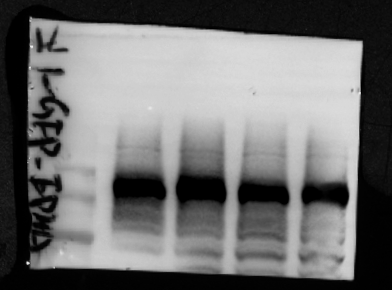

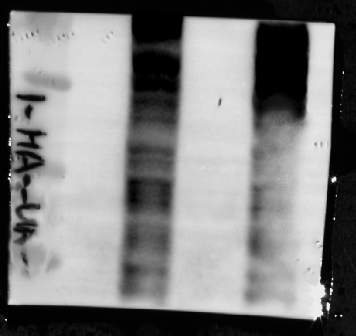


Lysis WB:GFP-EPHA2 Lysis WB:flag-MFSD4A Lysis WB:GAPDH


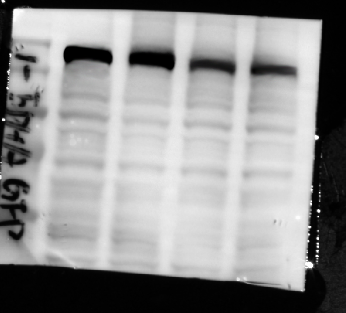

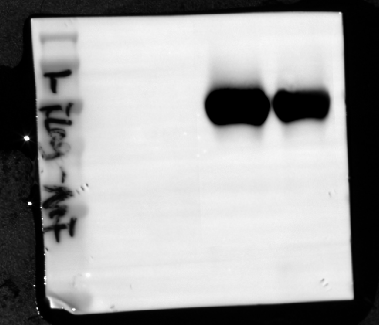

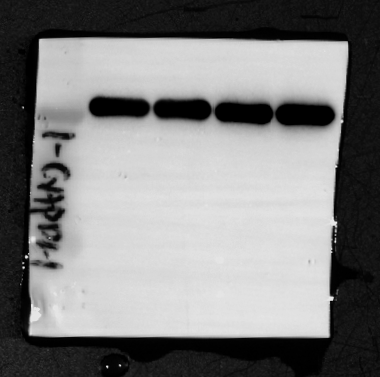


Figure 5H

Origin Figure


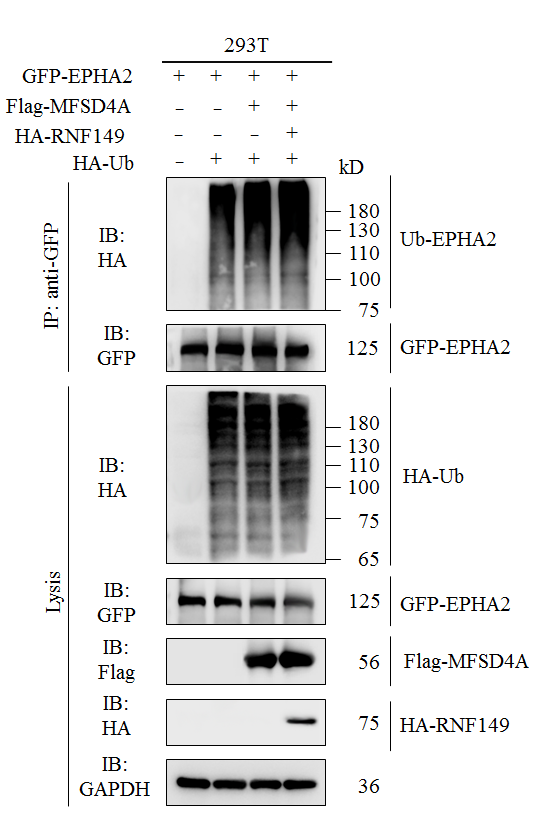


IP:GFP

WB:UB-EPHA2 WB:GFP-EPHA2 Lysis WB:HA-Ub


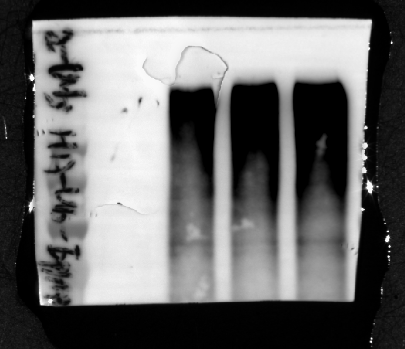

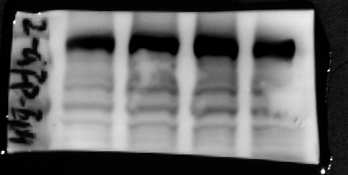

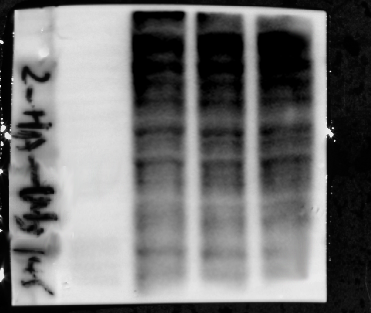


LysisWB:GFP-EPHA2 LysisWB:flag-MFSD4A LysisWB:HA-RNF149


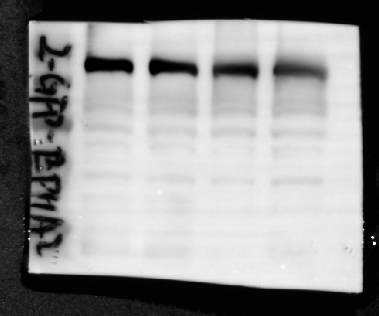

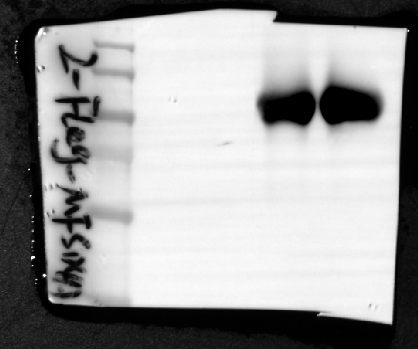

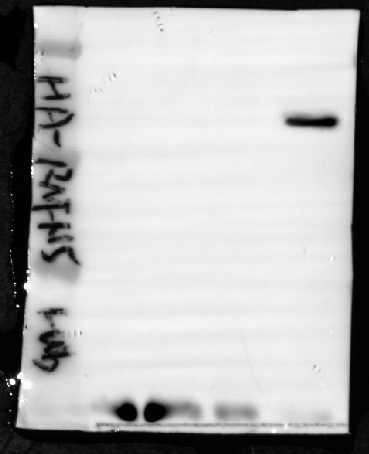


Lysis WB:GAPDH


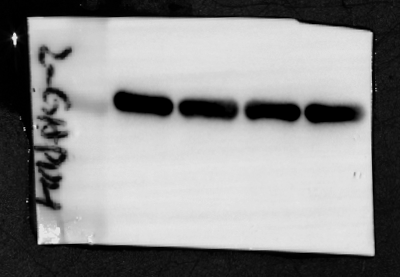


Figure 5I

Origin Figure


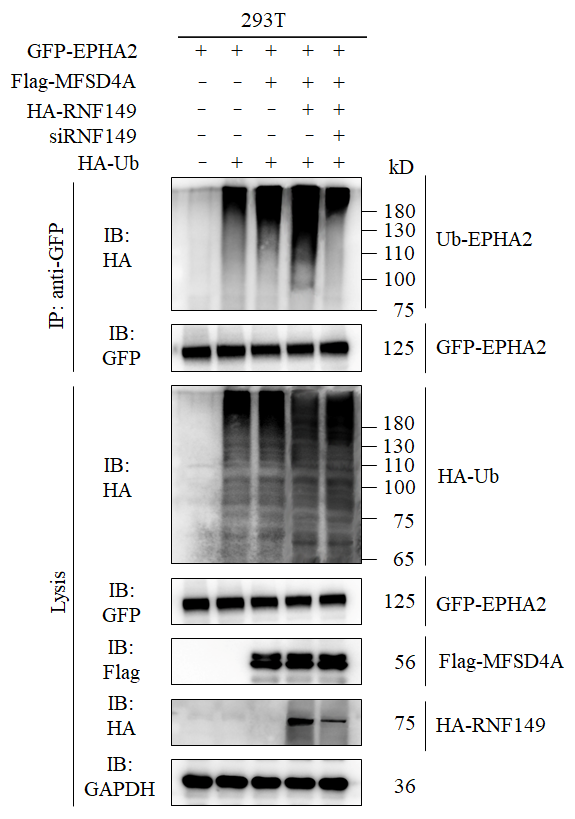


IP:GFP

WB:UB-EPHA2 WB:GFP-EPHA2 Lysis WB:HA-Ub


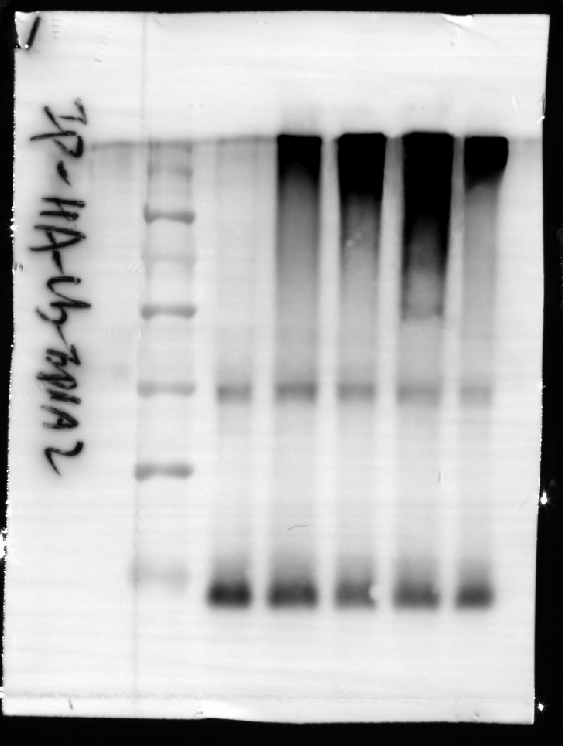


Lysis WB:GFP-EPHA2 Lysis WB:flag-MFSD4A Lysis WB:HA-RNF149

Lysis WB:GAPDH

Figure 6B

Origin Figure

MFSD4A EPHA2 GAPDH

Figure 6D

Origin Figure

MFSD4A EPHA2 GAPDH

Figure 6I

Origin Figure

MFSD4A

HONE1 SUNE1

EPHA2

HONE1 SUNE1

AKT

HONE1 SUNE1

P-AKT

HONE1 SUNE1

ERK

HONE1 SUNE1

P-EKR

HONE1 SUNE1

PI3K

HONE1 SUNE1

P-PI3K

HONE1 SUNE1

GAPDH

HONE1 SUNE1

Figure 6J

Origin Figure

MFSD4A

HONE1 SUNE1

EPHA2

HONE1 SUNE1

Vitmentin

HONE1 SUNE1

1. Cardherin

HONE1 SUNE1

1. Cardherin

HONE1 SUNE1

Slug

HONE1 SUNE1

Snail

HONE1 SUNE1

GAPDH

HONE1 SUNE1

Figure 6 O

MFSD4A

HONE1 SUNE1

EPHA2

HONE1 SUNE1

AKT

HONE1 SUNE1

P-AKT

HONE1 SUNE1

ERK

HONE1 SUNE1

P-ERK

HONE1 SUNE1

PI3K

HONE1 SUNE1

P-PI3K

HONE1 SUNE1

GAPDH

HONE1 SUNE1

Figure 6 P

Origin Figure

MFSD4A

HONE1 SUNE1

EPHA2

HONE1 SUNE

Vitmentin

HONE1 SUNE1

E-Cardherin

HONE1 SUNE1

N-Cardherin

HONE1 SUNE1

Slug

HONE1 SUNE1

Snail

HONE1 SUNE1

GAPDH

HONE1 SUNE1
